# Effectiveness of Astaxanthin as a Feed Supplement to Improve Growth Performance and Feed Utilization in Aquaculture Animals: A Meta-Analysis

**DOI:** 10.3390/antiox14050609

**Published:** 2025-05-19

**Authors:** Bowen Li, Chunxiu Chen, Xiaoqing Zhou, Huiru Liu, Zhixiong Zhou, Xiaoyu Wang, Jian Liang, Yongjun Guo, Shuang Liang

**Affiliations:** 1 Tianjin Key Laboratory of Aqua-Ecology and Aquaculture, College of Fisheries, Tianjin Agricultural University, Tianjin 300384, China; 2304028158@stu.tjau.edu.cn (B.L.); 2204010145@stu.tjau.edu.cn (X.Z.); liuhuiru@tjau.edu.cn (H.L.); zhouzhix@tjau.edu.cn (Z.Z.); wxy0618@tjau.edu.cn (X.W.); liangjian@tjau.edu.cn (J.L.); 2 Key Laboratory of Smart Breeding (Co-Construction by Ministry and Province, Ministry of Agriculture and Rural Affairs), Tianjin Agricultural University, Tianjin 300384, China; 3 Tianjin Fisheries Research Institute, Tianjin 300221, China; dtny@tjau.edu.cn

**Keywords:** astaxanthin, aquaculture animals, growth performance, feed utilization, meta-analysis

## Abstract

Aquaculture, a vital component of global food supply, faces challenges from environmental stressors that compromise aquatic animal health and productivity. Astaxanthin, a potent carotenoid antioxidant, has shown promise in enhancing growth and stress resilience in aquaculture species, yet its effects remain inconsistent across studies. This meta-analysis systematically evaluates the efficacy of dietary astaxanthin supplementation on growth, feed utilization, antioxidant capacity, and immune function in aquaculture animals. Following Preferred Reporting Items for Systematic Reviews and Meta-Analyses (PRISMA) guidelines, 64 studies (33 species, 964 comparisons) published prior to 2025 were analyzed using a random-effects model. Results demonstrated that astaxanthin significantly improved final body weight, weight gain rate, specific growth rate, survival rate, and protein efficiency ratio, while reducing feed conversion ratio. Additionally, it enhanced digestive enzyme activities, hepatopancreas antioxidant biomarkers, and immune parameters. The subgroup analysis revealed differences related to species, trophic level, and habitat, and estimated the optimal dose for key indicators. Despite heterogeneity and publication bias, adjusted effect sizes remained significant for most outcomes. These findings underscore astaxanthin’s potential as a multifunctional feed additive to promote sustainable aquaculture, though its efficacy depends on species, dosage, and environmental context, warranting further mechanistic and optimization studies.

## 1. Introduction

As a crucial pillar of global food production, the aquaculture industry has evolved into a sector of strategic importance and ecological benefits, driven by the dual forces of sustained population growth and surging protein demand. Aquatic foods provide high-quality protein, with global aquaculture production reaching 130.9 million tons in 2022, accounting for 15% of animal protein and 6% of total protein supplies worldwide. Its per capita consumption growth rate has surpassed that of meat and dairy products [1,2]. Against the background of wild fishery resources nearing exploitation limits, aquaculture has assumed multiple functions through extensive distribution networks and efficient circulation systems, ensuring food security, promoting poverty reduction, and driving socio-economic development [3,4,5]. However, behind rapid expansion lies multiple pressures, including high stocking densities, water quality fluctuations, and pathogenic challenges. These stressors make aquatic organisms more susceptible to diseases and stress-related responses, such as increased production of reactive oxygen species (ROS) and potential cellular oxidative damage, resulting in the death of aquaculture animals, ultimately leading to significant production declines [6].

Antioxidants, whether applied through environmental supplementation or dietary addition, can significantly mitigate hazards associated with environmental stressors in aquaculture farming. Carotenoids, as a class of natural functional pigments, not only serve coloring roles but also play critical functions as antioxidant supplements and multifunctional biological agents in aquaculture [7,8,9]. Among these, astaxanthin (3, 3′-dihydroxy-β, β-carotene-4, 4′-dione), the most representative carotenoid, is noted for its superior antioxidant properties, significantly outperforming traditional antioxidants such as β-carotene and vitamin E [10]. Consequently, it is widely utilized as an additive in aquatic feeds. This compound holds dual value in aquaculture nutrition: as a xanthophyll carotenoid, it directly enhances product quality by imparting commercially desirable pigmentation to fish skin and fillets, with its pigment deposition function being well-established [11]. Additionally, numerous studies demonstrate that astaxanthin, as a functional additive, not only improves aquatic organisms’ growth performance, survival rates, reproductive health, and egg quality optimization [12,13,14], but also enhances overall health by boosting endogenous enzymatic and non-enzymatic antioxidant systems [15,16], strengthening immune defense mechanisms, and exerting anti-inflammatory effects [17,18]. However, some research indicates neutral or even negative impacts of astaxanthin on growth performance in species such as discus fish (*Symphysodon* spp.) [19], striped catfish (*Pangasianodon hypophthalmus*) [17], and rainbow trout (*Oncorhynchus mykiss*) [20], with such variations potentially attributable to species-specific responses, dosage differences, or other confounding factors. Although there have been qualitative analyses (descriptive reviews) summarizing the effects and mechanisms of astaxanthin on the growth and health of farmed fish [21,22,23], no quantitative analyses (or meta-analyses) have been conducted to quantitatively evaluate the application effects of astaxanthin in aquatic animals.

Meta-analysis, a statistical analysis technique that synthesizes results from a group of independent studies related to a specific topic and design, enables comprehensive understanding of research questions and identification of sources of variability in outcomes [24]. In recent years, this methodology has seen preliminary applications in aquaculture nutrition, analyzing the impacts of aquatic feed composition variations [25,26] and water quality management [27] on aquatic species. However, no studies have yet systematically conducted meta-analyses to evaluate the effects of dietary astaxanthin on growth performance metrics, feed utilization efficiency, and survival rates in aquaculture animals. Given that astaxanthin has emerged as a research focus for advancing sustainable aquaculture development, with its application studies and feed innovations attracting dual attention from academic and industrial sectors, there is a critical need to extract and consolidate the available literature through meta-analytical approaches.

Therefore, the objective of this study is to systematically evaluate the effects of dietary astaxanthin on the growth and feed utilization of aquaculture animals across diverse studies, species, trophic level, and habitat through a meta-analysis.

## 2. Materials and Methods

This study strictly adhered to the Preferred Reporting Items for Systematic Reviews and Meta-Analyses (PRISMA) statement guidelines, conducting a comprehensive systematic literature search, screening, data extraction, and analysis to rigorously evaluate the effects of astaxanthin as a feed additive on growth performance in aquaculture animals. This project has been registered in the Open Science Framework (DOI: https://doi.org/10.17605/OSF.IO/P7W53).

### 2.1. Literature Search Strategy

To comprehensively gather the literature on the effects of astaxanthin as a feed additive on growth performance in aquaculture animals, two experienced researchers collaboratively developed and executed a literature search strategy. The search spanned the Web of Science, China National Knowledge Infrastructure (CNKI), and Google Scholar databases, encompassing English and Chinese research articles published prior to January 2025. Search terms were systematically formulated to address astaxanthin, aquaculture species, feed, and growth performance metrics, including: “fish” OR “crustacean” OR “invertebrate” OR “turtle” AND “astaxanthin” OR “astaxantin” AND “diet” OR “dietary” OR “feed” OR “aquafeed” AND “growth performance” OR “performance” OR “final body weight (FBW)” OR “protein efficiency ratio (PER)” OR “feed conversion ratio (FCR)” OR “feed efficiency (FE)” OR “weight gain rate (WGR)” OR “specific growth rate (SGR)” OR “survival rate (SR)”.

### 2.2. Study Selection

The retrieved literature citations and abstracts were imported into Zotero 6 software (Windows version, Corporation for Digital Scholarship, Fairfax, VA, USA) for preliminary processing, with duplicate entries removed using the built-in deduplication feature. Two researchers with extensive expertise in aquaculture nutrition then independently evaluated the titles and abstracts of the literature. During this phase, studies clearly unrelated to the research topic were excluded based on predefined screening criteria. For the potentially relevant literature identified in the initial screening, full texts were downloaded for in-depth review. The two researchers independently conducted detailed evaluations of the full texts, strictly applying inclusion and exclusion criteria to determine final eligible studies. In cases of disagreement between the researchers during the screening process, a third senior researcher was consulted to facilitate discussion, with decisions resolved through thorough analysis and consensus-building.

### 2.3. Inclusion/Exclusion Criteria

Studies were considered eligible if they met all the following inclusion criteria: (1) Research design and experimental protocols followed principles of randomized controlled trials; (2) Experimental subjects were aquaculture species; (3) Astaxanthin was supplemented in diets as a single compound or in combination, with no significant differences in diet formulation and nutritional composition between groups; (4) Reported one or more of the following response indicators in both control and astaxanthin-supplemented groups: final body weight (FBW), weight gain rate (WGR), specific growth rate (SGR), feed conversion ratio (FCR), feed efficiency (FE), protein efficiency ratio (PER), or survival rate (SR); (5) Provided means, replication numbers, and error estimates (e.g., standard deviation (SD), pooled standard error (PSE), or standard error (SE)) for each response parameter to calculate standardized mean differences and sampling variances. Studies were excluded if they met any of the following criteria: (1) Astaxanthin supplementation was not the sole variable; (2) Absence of SD, PSE, and SE data.

### 2.4. Data Extraction

Two researchers independently extracted the following descriptive information and outcome data from 64 eligible studies using a standardized extraction form: (1) Basic information (first author, title, publication year); (2) Study characteristics (cultured species, initial body weight, feeding trial duration, number of replicates per treatment group, number of animals per replicate); (3) Astaxanthin supplementation level; (4) Outcome data (mean values and error estimates for each indicator). When results of target metrics were presented in histograms, WebPlotDigitizer was employed to precisely extract mean values and corresponding error estimates. Additionally, habitat environment and trophic level information for aquatic species were obtained from the FishBase database (http://www.fishbase.se/, accessed on 20 January 2025) and previous studies, respectively. Discrepancies were resolved through discussion with a third researcher to reach consensus.

Growth parameters in the included studies were calculated as follows:

Weight gain rate (WGR, %) = 100 × (final body weight − initial body weight)/initial body weight;

Specific growth rate (SGR, %/d) = 100 × [ln (final body weight) − ln (initial body weight)]/days;

Feed conversion ratio (FCR) = feed intake/(final body weight − initial body weight);

Protein efficiency ratio (PER, %) = 100 × (body weight gain/protein intake);

Survival rate (SR, %) = 100 × (final fish number/initial fish number);

Some included studies utilized FE rather than FCR to evaluate aquaculture animals’ feed utilization. These studies were incorporated to expand the sample size for more precise meta-analytical outcomes. To better elucidate how astaxanthin influences growth performance and feed utilization in aquaculture animals, additional parameters were extracted for analysis, including digestive enzymes (amylase, lipase, protease), hepatopancreas antioxidant status (superoxide dismutase (SOD), catalase (CAT), malondialdehyde (MDA), total antioxidant capacity (T-AOC), glutathione (GSH), glutathione peroxidase (GSH-Px)), and immune-related markers (lysozyme, acid phosphatase (ACP), alkaline phosphatase (AKP), immunoglobulin (Ig), complement C3 (C3), and complement C4 (C4)).

### 2.5. Statistical Analysis

All statistical analyses and visualizations were conducted in the RStudio statistical program (R version 4.4.2). The metafor package [28] was employed to calculate effect sizes, sampling variances, and 95% confidence intervals (95% CI), as well as to perform analyses of heterogeneity, sensitivity, and publication bias. Forest plots and meta-regression diagrams were visualized using the ggplot2 package.

#### 2.5.1. Effect Size Calculation and Heterogeneity Test

This study employed standardized mean difference (SMD) effect sizes using Hedges’ *g* with 95% CI to evaluate differences between intervention and control groups, a metric suitable for integrating continuous variables with inconsistent measurement units across studies [29]. A random effects model was used to combine the effect size to deal with the heterogeneity of species differences and experimental cycles [30], which was realized by the rma function of the metafor package. Effect size interpretation followed established criteria: positive *g*-values (lower 95% CI > 0) indicated significant superiority of the intervention group, negative *g*-values (upper 95% CI < 0) indicated the opposite, while intervals encompassing zero denoted non-significant differences. Effect magnitudes were categorized as small (0.2 ≤ |*g*| < 0.5), medium (0.5 ≤ |*g*| < 0.8), and large (|*g*| ≥ 0.8) [31]. Heterogeneity was quantified using *I*^2^ statistics and Cochran’s Q-test, with classification thresholds: negligible (*I*^2^ ≤ 25%), low (25–50%), moderate (50–75%), and high (≥75%). Publication bias was assessed through funnel plot symmetry tests and Egger’s test (*p* < 0.05 indicating significance), supplemented by Trim and Fill method adjustments when required.

#### 2.5.2. Subgroup Analyses

Subgroup analyses were conducted to investigate sources of heterogeneity across studies and assess the influence of multiple factors on pooled effect sizes. To identify potential sources of variability, experimental animals were categorized into three subgroups prior to meta-analysis: (1) taxonomic groups (fish, crustacean, and sea cucumbers); (2) habitat types (marine, freshwater, and brackish water); and (3) trophic levels (low, medium, and high). Trophic level classifications followed established definitions from previous research: low trophic level (2 ≤ trophic level < 3), medium trophic level (3 ≤ trophic level < 4), and high trophic level (trophic level ≥ 4) [32]. Additionally, to evaluate the impact of astaxanthin supplementation levels on growth and feed utilization parameters, effect sizes between astaxanthin-supplemented groups and controls were calculated across different dosage ranges, enabling a preliminary assessment of optimal supplementation levels.

## 3. Results

### 3.1. Study Selection Process

**Figure 1:**
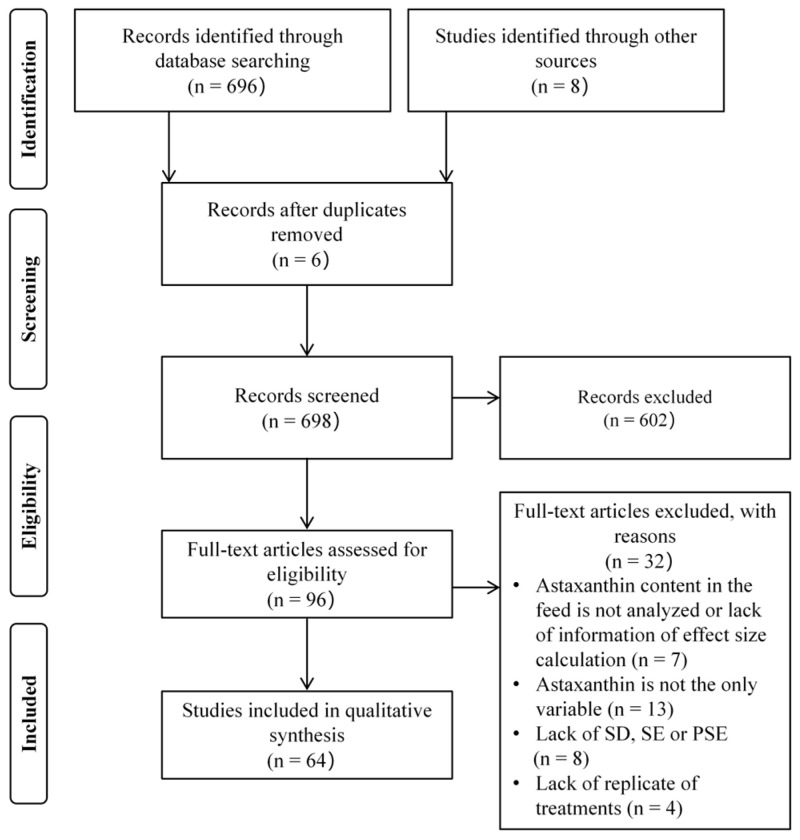
PRISMA flowchart used to select eligible studies for inclusion in a meta-analysis. SD, SE, and PSE are abbreviations for standard deviation, standard error, and pooled standard error, respectively.

The selection process of eligible articles for the meta-analysis is delineated in Figure 1. The database search retrieved 704 articles as of January 2025. After removing 6 duplicate records and excluding 602 articles through title and abstract screening, 96 articles underwent full-text evaluation. Among these, 32 articles were subsequently excluded due to failure to meet the predefined inclusion criteria. Ultimately, 64 high-quality articles were included in the meta-analysis.

### 3.2. Study Characteristics

The publication years of these studies spanned from 2005 to 2024, with the majority published within the past five years. A total of thirty-three aquaculture species from 64 articles were analyzed, including twenty-six fish species, six crustacean species, and one sea cucumber species. Among these species, seven were classified as low trophic level species, eighteen as medium trophic level species, and eight as high trophic level species. Astaxanthin supplementation levels ranged from 4 mg/kg to 4000 mg/kg. The meta-analysis incorporated 964 comparisons for growth and feed utilization parameters (*n* = 218 for FBW, *n* = 211 for WGR, *n* = 177 for SGR, *n* = 180 for FCR, *n* = 54 for PER, *n* = 124 for SR), 296 comparisons for hepatopancreas antioxidant parameters (*n* = 80 for SOD, *n* = 57 for CAT, *n* = 55 for MDA, *n* = 24 for GSH, *n* = 36 for GSH-Px, *n* = 44 for T-AOC), 42 comparisons for digestive enzymes (*n* = 14 for lipase, *n* = 14 for protease, *n* = 14 for amylase), and 134 comparisons for immune parameters (*n* = 46 for lysozyme, *n* = 27 for AKP, *n* = 24 for ACP, *n* = 17 for immunoglobulin, *n* = 11 for complement C3, *n* = 9 for complement C4). These comparisons were conducted between astaxanthin-supplemented groups and control groups. Table S1 present the general characteristics of studies included in the meta-analysis outcome metrics.

### 3.3. Effects of Astaxanthin on Growth Performance and Feed Utilization

**Figure 2:**
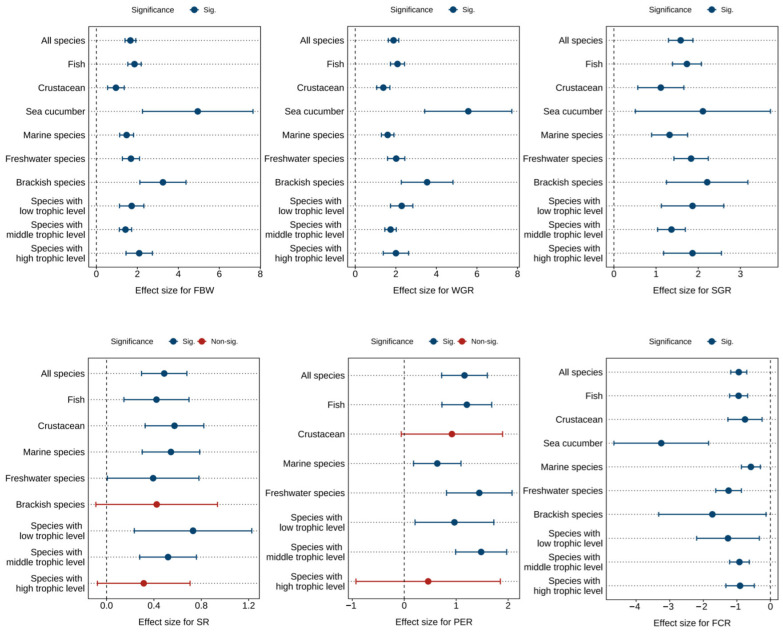
Pooled effects of adding astaxanthin to diet on growth performance (FBW, WGR, SGR, SR) and feed utilization (FCR, PER) with Hedge’s *g* (mean ± 95% CI). The 95% CI overlapping with 0 indicates no significant difference. FBW, final body weight; WGR, weight gain rate; SGR, specific growth rate; SR, survival rate; FCR, feed conversion ratio; PER, protein efficiency ratio.

Figure 2 and Table 1, Table 2 and Table 3 presents the combined effect sizes estimates of astaxanthin supplementation on growth performance (FBW, WGR, SGR, SR) and feed utilization (FCR, PER) in aquaculture animals. Overall, compared to the control group, astaxanthin supplementation significantly increased FBW (Hedges’ *g* = 1.66, 95% CI = 1.40 to 1.92, *p* < 0.0001), WGR (*g* = 1.89, 95% CI = 1.63 to 2.15, *p* < 0.0001), SGR (*g* = 1.58, 95% CI = 1.29 to 1.87, *p* < 0.0001), SR (*g* = 0.49, 95% CI = 0.30 to 0.68, *p* < 0.0001), and PER (*g* = 1.16, 95% CI = 0.72 to 1.60, *p* < 0.0001), while significantly reducing FCR (*g* = −0.94, 95% CI = −1.18 to −0.71, *p* < 0.0001).

Despite significant effects observed across all metrics, substantial heterogeneity was detected among studies (FBW: *I*^2^ = 72.51%; WGR: *I*^2^ = 68.41%; SGR: *I*^2^ = 72.73%; SR: *I*^2^ = 32.67%; FCR: *I*^2^ = 65.83%; PER: *I*^2^ = 63.50%). Subgroup analyses were consequently conducted to investigate sources of this heterogeneity.

Subgroup analysis revealed no detectable sources of heterogeneity for FBW, WGR, SGR, FCR, and PER (Figure 2). However, dietary astaxanthin supplementation showed no significant effects on SR in brackish water species and high trophic level species, nor on PER in crustaceans and high trophic level species.

Egger’s test (Table S13) and funnel plot (Figures S1–S6) suggested that there was potential publication bias in each index (*p* < 0.0001). Following the Trim and Fill adjustment, 51, 55, 43, 22, 13, and 36 missing studies were imputed for FBW, WGR, SGR, SR, PER, and FCR, respectively. The adjusted Hedges’ *g* values with 95% CI revealed: FBW (*g* = 0.96, 95% CI = 0.62 to 1.29, *p* < 0.0001), WGR (*g* = 1.19, 95% CI = 0.87 to 1.51, *p* < 0.0001), SGR (*g* = 0.87, 95% CI = 0.52 to 1.23, *p* < 0.0001), SR (*g* = 0.22, 95% CI = −0.0004 to 0.45, *p* = 0.0504), PER (*g* = 0.56, 95% CI = −0.004 to 1.13, *p* = 0.0501), and FCR (*g* = −0.42, 95% CI = −0.72 to −0.13, *p* = 0.0047). The effect sizes for FBW, WGR, SGR, and FCR remained statistically significant, while the effects of SR and PER were greatly affected by publication bias.

Furthermore, the analysis identified optimal response ranges for astaxanthin supplementation levels: 300 to 400 mg/kg for FBW and WGR, 100 to 200 mg/kg for SGR and SR, PER is greater than or equal to 1000 mg/kg, and 500 to 1000 mg/kg for FCR (Tables S2–S4).

### 3.4. Effects of Astaxanthin on Digestive Enzymes

**Figure 3:**
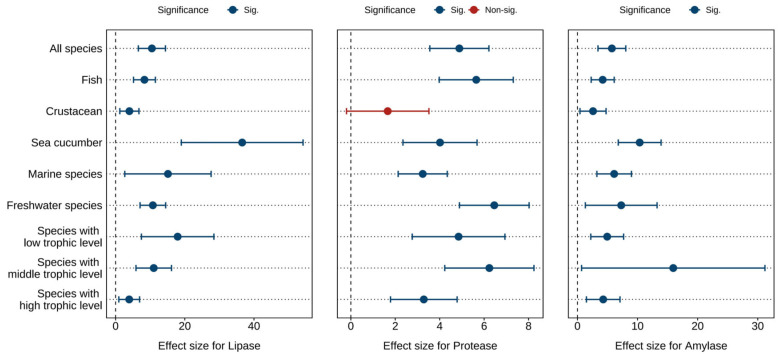
Pooled effects of adding astaxanthin to diet on digestive enzymes (lipase, protease, and amylase) with Hedge’s *g* (mean ± 95% CI). The 95% CI overlapping with 0 indicates no significant difference.

Figure 3 and Tables S5 and S6 displays the combined effect sizes estimates of astaxanthin supplementation on digestive enzymes (lipase, protease, and amylase) under a random effects model. The overall results demonstrate that, compared to the control group, astaxanthin supplementation significantly enhanced protease activity (*g* = 4.89, 95% CI = 3.56 to 6.22, *p* < 0.0001), lipase activity (*g* = 10.49, 95% CI = 6.57 to 14.42, *p* < 0.0001), and amylase activity (*g* = 5.73, 95% CI = 3.41 to 8.04, *p* < 0.0001). Substantial heterogeneity was observed across studies, with high heterogeneity for lipase (*I*^2^ = 88.14%) and amylase (*I*^2^ = 85.21%), and moderate heterogeneity for protease (*I*^2^ = 56.11%).

According to the Egger’s test (*p* < 0.0001) (Table S13) and funnel plot (Figures S7–S9), there was potential publication bias in all indicators. After applying the Trim and Fill adjustment, five, five, and four missing studies were imputed for protease, lipase, and amylase, respectively. The adjusted Hedges’ *g* values with 95% CI showed: protease (*g* = 3.74, 95% CI = 2.14 to 5.33, *p* < 0.0001), lipase (*g* = 6.93, 95% CI = 0.93 to 12.93, *p* = 0.0236), and amylase (*g* = 4.03, 95% CI = 0.35 to 7.70, *p* = 0.0318). These results indicate that publication bias did not significantly alter the pooled effect sizes for protease, lipase, or amylase.

### 3.5. Effects of Astaxanthin on Hepatopancreas Antioxidant Biomarkers

**Figure 4:**
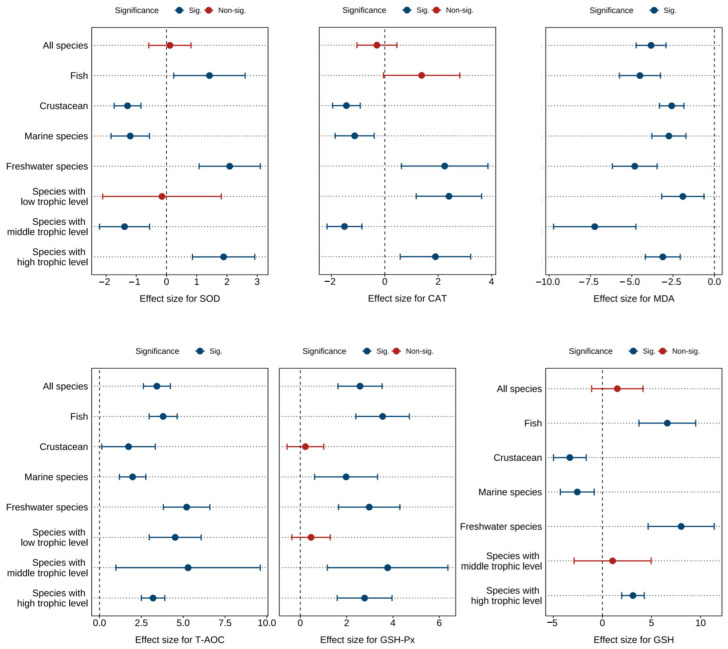
Pooled effects of adding astaxanthin to diet on hepatopancreas antioxidant biomarkers (SOD, CAT, MDA, T-AOC, GSH-Px, GSH) with Hedge’s *g* (mean ± 95% CI). The 95% CI overlapping with 0 indicates no significant difference. SOD, superoxide dismutase; CAT, catalase; MDA, malondialdehyde; T-AOC, total antioxidant capacity; GSH-Px, glutathione peroxidase; GSH, glutathione.

Figure 4 and Tables S7–S9 show the combined effect sizes of astaxanthin supplementation on hepatopancreas antioxidant biomarkers (SOD, CAT, MDA, T-AOC, GSH, GSH-Px) under the random effects model. The effect sizes demonstrated that dietary astaxanthin significantly enhanced hepatopancreas T-AOC activity (*g* = 3.43, 95% CI = 2.62 to 4.24, *p* < 0.0001) and GSH-Px activity (*g* = 2.58, 95% CI = 1.63 to 3.53, *p* < 0.0001), while significantly reducing hepatopancreas MDA content (*g* = −3.83, 95% CI = −4.73 to −2.93, *p* < 0.0001). However, no significant differences were observed between astaxanthin-supplemented and control groups for SOD (*g* = 0.11, 95% CI = −0.59 to 0.81, *p* = 0.7497), CAT (*g* = −0.30, 95% CI = −1.05 to 0.45, *p* = 0.4368), and GSH levels (*g* = 1.52, 95% CI = −1.10 to 4.13, *p* = 0.2553). Based on the *I*^2^ statistic, high heterogeneity was detected across all biomarkers (79.10% to 95.06%).

Subgroup analysis revealed differential effects of astaxanthin across subgroups. However, consistent significant improvements were observed in SOD, CAT, MDA, T-AOC, GSH, and GSH-Px within freshwater species subgroups and high-trophic-level species subgroups (Figure 4).

Egger’s test indicated potential publication bias across all biomarkers (*p* < 0.0001) (Table S13). Following the Trim and Fill adjustment (Figures S10–S15), four, two, fifteen, fifteen, and nine missing studies were imputed for SOD, CAT, MDA, T-AOC, and GSH-Px, respectively. The adjusted Hedges’ *g* values with 95% CI were: SOD (*g* = 0.46, 95% CI = −0.31 to 1.23, *p* = 0.2445), CAT (*g* = −0.12, 95% CI = −0.89 to 0.65, *p* = 0.7520), MDA (*g* = −2.47, 95% CI = −3.67 to −1.27, *p* < 0.0001), T-AOC (*g* = 2.08, 95% CI = 0.94 to 3.22, *p* = 0.0003), and GSH-Px (*g* = 1.55, 95% CI = 0.42 to 2.68, *p* = 0.0069). GSH was not found to be missing. Consequently, publication bias did not significantly affect the pooled effect sizes for SOD, CAT, MDA, T-AOC, GSH, or GSH-Px.

### 3.6. Effects of Astaxanthin on Immune-Related Parameters

**Figure 5:**
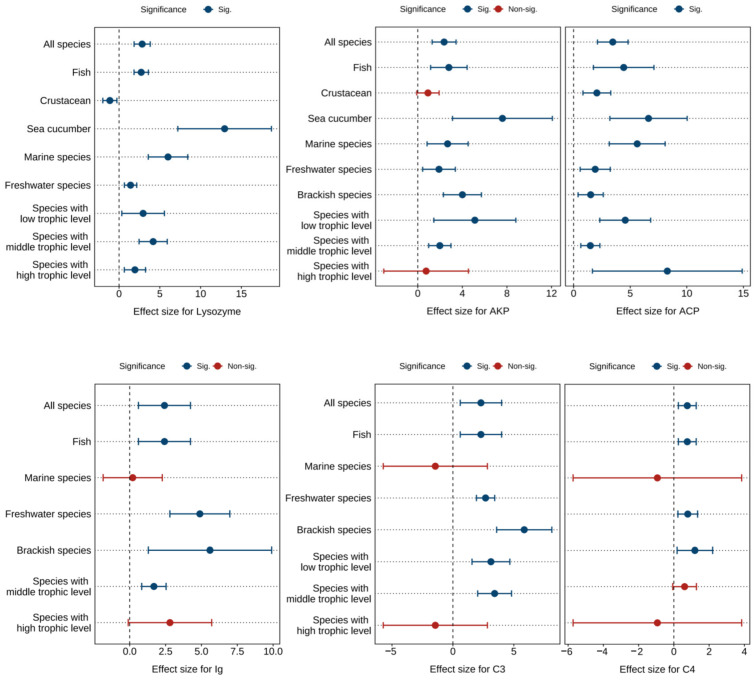
Pooled effects of adding astaxanthin to diet on immune parameters (lysozyme, AKP, ACP, immunoglobulin, complement C3, complement C4) with Hedge’s *g* (mean ± 95% CI). The 95% CI overlapping with 0 indicates no significant difference. AKP, alkaline phosphatase; ACP, acid phosphatase; Ig, immunoglobulin; C3, complement C3; C4, complement C4.

Figure 5 and Tables S10–S12 present the combined effect sizes of astaxanthin supplementation on immune parameters (lysozyme, immunoglobulin, complement C3, complement C4, AKP, ACP) under a random effects model. The overall effect sizes demonstrated that dietary astaxanthin significantly enhanced lysozyme activity (*g* = 2.83, 95% CI = 1.85 to 3.82, *p* < 0.0001), immunoglobulin levels (*g* = 2.42, 95% CI = 0.61 to 4.24, *p* = 0.0088), complement C3 levels (*g* = 2.30, 95% CI = 0.60 to 4.00, *p* = 0.0079), complement C4 levels (*g* = 0.76, 95% CI = 0.25 to 1.27, *p* = 0.0033), AKP activity (*g* = 2.37, 95% CI = 1.29 to 3.44, *p* < 0.0001), and ACP activity (*g* = 3.46, 95% CI = 2.11 to 4.81, *p* < 0.0001). Based on the *I*^2^ statistic, high heterogeneity (86.06% to 89.93%) was detected across all biomarkers except complement C4, which exhibited no heterogeneity (0%).

Subgroup analysis revealed differential effects of astaxanthin on immune parameters in crustacean, marine species, and high-trophic-level species subgroups, while consistent significant improvements were observed across other subgroups (Figure 5).

Egger’s test showed that there was potential publication bias in each index (*p* < 0.0001) (Table S13). Following the Trim and Fill adjustment (Figures S16–S21), twelve, five, three, two, seven, and seven missing studies were imputed for lysozyme, immunoglobulin, complement C3, complement C4, AKP, and ACP, respectively. The adjusted Hedges’ *g* values with 95% CI were: lysozyme (*g* = 1.55, 95% CI = 0.07 to 3.03, *p* = 0.0403), immunoglobulin (*g* = 0.85, 95% CI = −1.19 to 2.88, *p* = 0.4146), complement C3 (*g* = 1.31, 95% CI = −0.45 to 3.07, *p* = 0.1451), complement C4 (*g* = 0.62, 95% CI = 0.15 to 1.09, *p* = 0.0104), AKP (*g* = 1.31, 95% CI = −0.14 to 2.76, *p* = 0.0755), and ACP (*g* = 1.89, 95% CI = 0.16 to 3.76, *p* = 0.0481). Consequently, publication bias did not significantly affect the combined effect sizes for lysozyme, complement C4, and ACP, but exerted notable impacts on immunoglobulin, complement C3, and AKP.

## 4. Discussion

### 4.1. Effects of Astaxanthin on Growth Performance and Feed Utilization of Aquaculture Animals

This study comprehensively demonstrates that dietary astaxanthin supplementation significantly improves FBW (*g* = 1.66), WGR (*g* = 1.89), SGR (*g* = 1.58), FCR (*g* = −0.94), and PER (*g* = 1.16) in aquaculture species. Despite the high heterogeneity, it is undeniable that the average effect sizes of FBW, WGR, SGR, PER, and FCR in the astaxanthin-supplemented group compared with the control group are all beneficial for aquaculture animals. This indicates that, on average, adding astaxanthin to the feed can significantly improve the growth performance and feed utilization rate of aquaculture animals.

The growth-promoting effects of astaxanthin supplementation may be attributed to its multifaceted physiological roles: (1) The intake of astaxanthin can lead to the improvement of intestinal morphology (villus length, muscle thickness) in aquatic animals, thereby enhancing the absorption of nutrients [19]. In aquatic animals, the intestine is the main organ for digesting and absorbing nutrients from food. Therefore, digestive function is closely related to the development of the intestine [33]. Some studies have reported that increasing the length of intestinal villi can expand the intestinal surface area to enhance digestive and absorptive functions [34,35]. The increase in muscle thickness is positively correlated with intestinal peristalsis ability. This structural optimization is conducive to the transportation and absorption of nutrients [36]. The intake of astaxanthin can also increase the density of goblet cells in the intestinal tract and pyloric diverticulum of fish, thereby protecting the mucosal layer from harmful substances [37]. This has been confirmed in numerous experiments on species such as white shrimp (*Litopenaeus vannamei*) [12], largemouth bass (*Micropterus salmoides*) [13], rainbow trout (*Oncorhynchus mykiss*) [14], and golden pompano (*Trachinotus ovatus*) [38]. (2) Digestive enzymes (protease, lipase and amylase) can break down proteins, fats, and starches into small molecules that can be absorbed. The activity of digestive enzymes can directly or indirectly determine the maintenance status of nutrients [39]. This study demonstrates that adding astaxanthin to the feed can increase the activities of lipase (*g* = 10.49), protease (*g* = 4.89), and amylase (*g* = 5.73) in the aquaculture animals’ bodies. This is consistent with the experimental results of species such as coral trout (*Plectropomus leopardus*) [40], kuruma shrimp (*Marsupenaeus japonicus*) [41], sea cucumber (*Apostichopus japonicas*) [42], and crucian carp (*Carassius auratus*) [43]. (3) Studies have indicated that the synthesis of certain substances in animal organisms requires NADPH to provide reducing power, such as the synthesis of cholesterol, amino acids, and nucleic acids, among others [44]. Astaxanthin exhibits exceptional antioxidant activity, which can inhibit NADPH reductase activity and elevate NADPH levels, thereby promoting the biosynthesis of proteins, lipids, and carbohydrates [45]. Furthermore, leveraging its potent antioxidant properties, astaxanthin modulates microbial balance by maintaining a reductive intestinal environment. This regulatory mechanism not only selectively inhibits the proliferation of aerobic bacteria and reduces the production of exogenous digestive enzymes, but also activates the gene expression of the digestive enzyme system within the host, achieving bidirectional regulation of digestion and metabolism [19]. (4) Studies have revealed that astaxanthin activates peroxisome proliferator-activated receptor α (PPARα), which significantly downregulates the mRNA expression of peroxisome proliferator-activated receptor γ (PPARγ), the primary adipogenic transcription factor. This mechanism effectively reduces hepatic lipid accumulation in mice, demonstrating astaxanthin’s lipid-lowering properties [46,47]. The addition of astaxanthin to the feed significantly reduced the fat content in the bodies of rainbow trout (*Oncorhynchus mykiss*) [14] and red porgy (*Pagrus pagrus*) [48]. This effect may be attributed to the capacity of dietary astaxanthin to enhance lipid metabolism in aquatic species, coupled with its potential protein-sparing function, which thereby provides more energy for the growth of aquaculture animals.

### 4.2. Effects of Astaxanthin on Survival Rate, Antioxidant Capacity, and Immune Function in Aquaculture Animals

Dietary astaxanthin supplementation significantly improved the survival rate of aquaculture animals (*g* = 0.49, 95% CI = 0.30–0.68, *p* < 0.0001). This may be related to the findings from this study that dietary supplementation of astaxanthin significantly improved hepatopancreas antioxidant biomarkers (MDA, T-AOC, GSH, GSH-Px) and immune-related parameters (lysozyme, immunoglobulin, complement C3, complement C4, AKP, ACP) in aquaculture animals [16,49].

#### 4.2.1. Effects of Astaxanthin on Antioxidant Capacity of Aquaculture Animals

As a strong antioxidant found in nature, astaxanthin has been proved to have antioxidant activity in many studies [50,51,52]. Oxidative stress is a key determinant of fish health [53]. Oxidative stress refers to a state of imbalance between the production of harmful ROS and the ability of organisms to alleviate their harmful effects through antioxidants [54]. Oxidative stress is a pathological state of the imbalance of redox homeostasis in the body, which is manifested as the imbalance between the excessive production of intracellular ROS and the scavenging ability of endogenous antioxidant defense system [55]. Under normal physiological conditions, the body effectively regulates the level of ROS through the antioxidant enzyme network and DNA repair mechanisms to maintain cellular homeostasis. This balance plays a crucial role in maintaining the overall health and production efficiency of aquaculture animals [23]. However, when this balance system is disrupted, the excessive ROS, due to its strong oxidizing properties, can undergo irreversible oxidative reactions with biological macromolecules such as proteins, lipids, and nucleic acids, leading to protein denaturation, lipid peroxidation, and DNA strand breaks, and subsequently triggering the occurrence and development of various diseases [56,57].

In this study, the addition of astaxanthin to the feed significantly increased the levels of T-AOC (*g* = 3.43) in the hepatopancreas of aquaculture animals, enhanced the activity of GSH-Px (*g* = 2.58), and significantly reduced the content of MDA (*g* = −3.83). MDA is the main product of polyunsaturated fatty acid peroxidation. As a biomarker of oxidative stress, it reflects the degree of lipid peroxidation caused by free radicals and mediates cellular damage [58]. GSH-Px can eliminate the accumulated peroxylipids and MDA produced within cells, thereby protecting cells from oxidative damage [56]. T-AOC is a comprehensive indicator for evaluating the antioxidant capacity of an animal’s body. It can reflect the overall ability of the body to eliminate ROS. The higher its value is, the higher the total antioxidant level composed of various antioxidant substances and antioxidant enzymes is [59,60]. This is consistent with the experimental results of rainbow trout (*Oncorhynchus mykiss*) [61], snakehead (*Channa argus*) [62], Giant grouper (*Epinephelus lanceolatus*) [63], Chinese mitten crab (*Eriocheir sinensis*) [64], golden pompano (*Trachinotus ovatus*) [65], discus fish (*Symphysodon* spp.) [66], and coral trout (*Plectropomus leopardus*) [40].

It is worth noting that SOD and CAT are also regarded as important indicators for evaluating the antioxidant status. In biological systems, SOD and CAT are significant components of antioxidant enzymes. SOD plays a crucial role in eliminating harmful ROS and preventing oxidative stress [67]. CAT is one of the key enzymes in the ROS scavenging metabolic pathway. CAT rapidly eliminates H_2_O_2_ through disproportionation reaction, generating H_2_O and O_2_ [68]. GSH is a tripeptide composed of glutamic acid, cysteine, and glycine through peptide bond condensation. It can directly neutralize free radicals and promote the activity of GSH-Px [69]. However, in this study, supplementation of astaxanthin did not significantly enhance the activities of SOD (−0.59 to 0.81), CAT (−1.05 to 0.45), and GSH (−1.10 to 4.13) in the hepatopancreas of aquaculture animals. This might be because astaxanthin, as a non-enzymatic system component, participates in the antioxidant process and effectively eliminates excessive oxygen free radicals before the endogenous antioxidant enzymes are activated [70], thereby reducing the reaction substrates of antioxidant enzymes and ultimately leading to a decrease in antioxidant enzyme activity [15,71]; this is consistent with the experimental results of species such as golden pompano (*Trachinotus ovatus*) [16,65], Chinese mitten crab (*Eriocheir sinensis*) [64], and rainbow trout (*Oncorhynchus mykiss*) [15,20], where the activities of SOD or CAT decreased while T-AOC levels increased after supplementation with astaxanthin.

In conclusion, supplementing feed with astaxanthin can enhance the antioxidant defense capacity of the organism, strengthen its resistance to oxidative stress, inhibit the inflammatory and apoptotic cascade reactions, and improve the survival rate of aquaculture animals.

#### 4.2.2. Effects of Astaxanthin on Immune Function of Aquaculture Animals

In addition to its antioxidant capacity, multiple studies have demonstrated that astaxanthin can improve the immune function of the body through various mechanisms, including cellular immunity and humoral immunity of the body [72,73,74]. Astaxanthin can not only maintain the redox balance within lymphocytes and neutrophils by quenching free radicals to restore the normal phagocytic function of immune cells, but also enhance the body’s ability to resist exogenous pathogen invasion by stimulating the mitotic division of T cells and B cells and promoting the proliferation of PBMC and the differentiation of peripheral B lymphocytes into antibodies [75,76,77,78].

In this study, the addition of astaxanthin to the feed significantly enhanced the activities of lysozyme (*g* = 2.83), AKP (*g* = 2.37), and ACP (*g* = 3.46) of aquaculture animals, as well as the levels of immunoglobulin (*g* = 2.42), complement C3 (*g* = 2.30), and complement C4 (*g* = 0.76). This is in line with the research conclusions of species such as white shrimp (*Litopenaeus vannamei*) [12], striped catfish (*Pangasianodon hypophthalmus*) [17], crucian carp (*Carassius auratus*) [43], common carp (*Cyprinus carpio*) [18], largemouth bass (*Micropterus salmoides*) [13], and snakehead (*Channa argus*) [62]. The lysozyme in the blood is a marker that can be used to assess the innate immune response of fish. Lysozyme can hydrolyze the cell walls of bacteria, thereby exerting the function of killing bacteria [79]. AKP and ACP are important metabolic regulatory enzymes in organisms, involved in phosphate group transfer reactions and phosphorus metabolism. They can eliminate invasive pathogens and promote phagocytosis and the degradation ability of foreign substances [80]. Immunoglobulin is an antibody protein secreted by plasma cells that can bind to specific antigens. It has powerful bactericidal, complement activation, immune modulation, and agglutination effects. An increase in immunoglobulin levels indicates enhanced immune capacity of the body [81]. Complement C3 and C4 are the most abundant complement components in serum. As the core mediators of humoral immune response and inflammatory response, they play a role in sterilization and immune regulation after activation by assisting specific antibodies, mediating bacteriolysis and hemolysis [82]. The non-significant results presented in the subgroups might be attributed to the small sample size.

Therefore, supplementing astaxanthin in the feed can also improve the immune function of aquaculture animals to a certain extent, enhance their disease resistance, and increase their survival rate.

### 4.3. Limitaions

There are some limitations in this study. First, despite conducting subgroup analyses, substantial heterogeneity persisted in some subgroups due to the diverse range of species in aquaculture systems. Secondly, many previous studies have explored the coloration effect of astaxanthin on aquaculture animals. Atlantic salmon (*Salmo salar*), as a representative of brightly colored fish, has been extensively studied for the retention rate of astaxanthin in its body and its impact on flesh color. However, due to the fact that this study mainly evaluated the effect of astaxanthin as a feed additive for aquaculture animals on their growth performance and the limitations of the search term, studies on Atlantic salmon were not included in this research. The specific reasons for exclusion included (but were not limited to) the absence of a blank control in randomized trials, lack of a standardized feeding system, and failure to report means, repetitions, and error estimates. In addition, we conducted additional searches in Web of Science using the terms (“Salmo” AND “astaxanthin” OR “astaxantin”) and (“charr” AND “astaxanthin” OR “astaxantin”), obtaining 338 and 34 studies respectively. After initial screening and downloading the full texts for in-depth evaluation, no studies met the inclusion criteria of this article.

Nevertheless, this study incorporated multiple investigations examining astaxanthin’s effects as an aquafeed additive on growth performance in salmon families. Among the 64 articles ultimately included in the analysis, 11 specifically addressed astaxanthin’s impacts on salmonid species, including 10 studies on rainbow trout (*Oncorhynchus mykiss*) and 1 study on Black Sea trout (*Salmo labrax Pallas*, 1814). After incorporating these studies, this research also to some extent explains the impact of astaxanthin on the growth performance of salmonid fish.

This article has already investigated the impact of astaxanthin on the growth performance of salmonid fish. Future meta-analyses on the effects of astaxanthin on aquaculture animals should consider targeted investigations for specific species or functions, such as studying the impact of astaxanthin on specific growth qualities of salmonids (e.g., flesh color), to better clarify and expand the role of astaxanthin in aquaculture practices.

## 5. Conclusions

Given the multi-faceted physiological functions of astaxanthin, the current quantitative meta-analysis indicates that adding astaxanthin to the feed significantly improves the FBW, WGR, SGR, SR, and FCR of aquaculture animals, while reducing the FCR. Furthermore, supplementation with astaxanthin has exerted positive effects on the digestive, antioxidant and immune functions of aquaculture animals. Therefore, astaxanthin is a promising feed additive for promoting the growth of aquaculture animals, and it is of great significance for sustainable aquaculture. However, the optimal addition amount of astaxanthin and the effect of astaxanthin on the growth performance of aquatic animals are closely related to the developmental stage of the animals, the breeding environment, species differences, the source of astaxanthin, and the duration of feeding. Further research is needed to explore the mechanism of the impact of astaxanthin on the growth of aquatic animals in the future.

## Figures and Tables

**Table 1:** Effect size calculation for final body weight and weight gain rate comparisons based on random-effect model.

	Final Body Weight							Weight Gain Rate						
	*k*	*I* ^2^	Hedges’ *g* Value	SE	Ci.Lb	Ci.Ub	*p*-Value	*k*	*I* ^2^	Hedges’ *g* Value	SE	Ci.Lb	Ci.Ub	*p*-Value
All species	218	72.51	1.6611	0.1345	1.3976	1.9247	<0.0001	211	68.41	1.8872	0.1317	1.6290	2.1454	<0.0001
***Subgroups***														
*Species category*														
Fish	157	74.00	1.8612	0.1663	1.5353	2.1872	<0.0001	153	74.25	2.0830	0.1768	1.7365	2.4295	<0.0001
Crustacean	55	61.58	0.9545	0.2070	0.5488	1.3602	<0.0001	55	38.37	1.3843	0.1659	1.0592	1.7094	<0.0001
Sea cucumber	6	76.69	4.9470	1.3762	2.2496	7.6443	0.0003	3	0	5.5728	1.0956	3.4254	7.7202	<0.0001
*habitat*														
Marine	115	68.99	1.4755	0.1729	1.1366	1.8143	<0.0001	104	58.97	1.5974	0.1582	1.2874	1.9073	<0.0001
Freshwater	93	74.77	1.6874	0.2136	1.2687	2.1061	<0.0001	97	73.28	2.0181	0.2171	1.5926	2.4436	<0.0001
Brackish	10	51.88	3.2489	0.5746	2.1227	4.3750	<0.0001	10	58.79	3.5435	0.6505	2.2685	4.8185	<0.0001
* Trophic level*														
Low trophic level	26	52.92	1.7235	0.3033	1.1290	2.3180	<0.0001	18	11.18	2.2895	0.2810	1.7388	2.8401	<0.0001
Middle trophic level	126	65.70	1.4206	0.1534	1.1198	1.7213	<0.0001	132	58.71	1.7410	0.1440	1.4587	2.0233	<0.0001
High trophic level	66	83.10	2.0906	0.3282	1.4473	2.7338	<0.0001	61	81.30	2.0050	0.3185	1.3808	2.6292	<0.0001

*k*, sample size; *I*^2^ percentage changes resulting from heterogeneity among different studies; SE, standard error; Ci.Lb, confidence interval lower bound; Ci.Ub, confidence interval upper bound.

**Table 2:** Effect size calculation for specific growth rate and survival rate comparisons based on random-effect model.

	Specific Growth Rate							Survival Rate						
	*k*	*I* ^2^	Hedges’ *g* Value	SE	Ci.Lb	Ci.Ub	*p*-Value	*k*	*I* ^2^	Hedges’ *g* Value	SE	Ci.Lb	Ci.Ub	*p*-Value
All species	177	72.73	1.5832	0.1479	1.2934	1.8730	<0.0001	124	32.67	0.4870	0.0977	0.2954	0.6785	<0.0001
***Subgroups***														
* Species category*														
Fish	121	71.58	1.7314	0.1744	1.3895	2.0733	<0.0001	70	43.07	0.4214	0.1401	0.1468	0.6961	0.0026
Crustacean	50	72.41	1.1124	0.2788	0.5660	1.6589	<0.0001	54	6.54	0.5737	0.1263	0.3260	0.8213	<0.0001
Sea cucumber	6	70.20	2.1105	0.8171	0.5089	3.7120	0.0098	NA	NA	NA	NA	NA	NA	NA
* habitat*														
Marine	95	75.68	1.3197	0.2179	0.8926	1.7468	<0.0001	78	30.79	0.5438	0.1238	0.3011	0.7865	<0.0001
Freshwater	72	67.92	1.8303	0.2080	1.4227	2.2380	<0.0001	36	44.63	0.3934	0.1974	0.0065	0.7803	0.0463
Brackish	10	52.73	2.2123	0.4922	1.2476	3.1770	<0.0001	10	0	0.4234	0.2619	−0.0900	0.9368	0.1060
* Trophic level*														
Low trophic level	21	61.16	1.8648	0.3773	1.1253	2.6043	<0.0001	18	0	0.7304	0.2531	0.2343	1.2264	0.0039
Middle trophic level	110	66.86	1.3649	0.1675	1.0365	1.6933	<0.0001	72	31.27	0.5193	0.1221	0.2800	0.7585	<0.0001
High trophic level	46	80.63	1.8640	0.3499	1.1781	2.5498	<0.0001	34	44.07	0.3140	0.1996	−0.0771	0.7052	0.1156

*k*, sample size; *I*^2^ percentage changes resulting from heterogeneity among different studies; SE, standard error; Ci.Lb, confidence interval lower bound; Ci.Ub, confidence interval upper bound; NA, not applicable.

**Table 3:** Effect size calculation for feed conversion ratio and protein efficiency ratio comparisons based on random-effect model.

	Feed Conversion Ratio							Protein Efficiency Ratio						
	*k*	*I* ^2^	Hedges’ *g* Value	SE	Ci.Lb	Ci.Ub	*p*-Value	*k*	*I* ^2^	Hedges’ *g* Value	SE	Ci.Lb	Ci.Ub	*p*-Value
All species	180	65.83	−0.9432	0.1215	−1.1813	−0.7051	<0.0001	54	63.50	1.1603	0.2244	0.7205	1.6001	<0.0001
*** Subgroups***														
* Species category*														
Fish	150	67.57	−0.9480	0.1373	−1.2172	−0.6788	<0.0001	51	66.88	1.2052	0.2446	0.7257	1.6847	<0.0001
Crustacean	27	55.20	−0.7573	0.2597	−1.2664	−0.2483	0.0035	3	0	0.9177	0.4977	−0.0578	1.8933	0.0652
Sea cucumber	3	0	−3.2515	0.7193	−4.6613	−1.8416	<0.0001	NA	NA	NA	NA	NA	NA	NA
* habitat*														
Marine	79	49.72	−0.5822	0.1441	−0.8647	−0.2998	<0.0001	14	0	0.6359	0.2332	0.1789	1.0930	0.0064
Freshwater	91	71.97	−1.2465	0.1950	−1.6287	−0.8643	<0.0001	40	73.60	1.4449	0.3219	0.8139	2.0759	<0.0001
Brackish	10	83.32	−1.7292	0.8158	−3.3281	−0.1303	0.0340	NA	NA	NA	NA	NA	NA	NA
* Trophic level*														
Low trophic level	19	75.27	−1.2641	0.4761	−2.1972	−0.3309	0.0079	5	0	0.9665	0.3870	0.2080	1.7250	<0.0001
Middle trophic level	93	56.70	−0.9232	0.1495	−1.2162	−0.6303	<0.0001	36	55.85	1.4807	0.2506	0.9896	1.9718	<0.0001
High trophic level	68	72.24	−0.9044	0.2156	−1.3269	−0.4818	<0.0001	13	82.90	0.4602	0.7095	−0.9304	1.8509	0.5166

*k*, sample size; *I*^2^ percentage changes resulting from heterogeneity among different studies; SE, standard error; Ci.Lb, confidence interval lower bound; Ci.Ub, confidence interval upper bound; NA, not applicable.

## Data Availability

The data that support the findings of this study are available on request from the corresponding author.

## Supplementary Materials

The following supporting information can be downloaded at: https://www.mdpi.com/article/10.3390/antiox14050609/s1, Figure S1: Evidence of publication (reporting) bias for final body weight (FBW); Figure S2: Evidence of publication (reporting) bias for weight gain rate (WGR).; Figure S3: Evidence of publication (reporting) bias for specific growth ratio (SGR); Figure S4: Evidence of publication (reporting) bias for survival rate (SR); Figure S5: Evidence of publication (reporting) bias for protein efficiency ratio (PER); Figure S6: Evidence of publication (reporting) bias for feed conversion ratio (FCR); Figure S7: Evidence of publication (reporting) bias for lipase; Figure S8: Evidence of publication (reporting) bias for protease; Figure S9: Evidence of publication (reporting) bias for amylase; Figure S10: Evidence of publication (reporting) bias for SOD; Figure S11: Evidence of publication (reporting) bias for CAT; Figure S12: Evidence of publication (reporting) bias for MDA; Figure S13: Evidence of publication (reporting) bias for T-AOC; Figure S14: Evidence of publication (reporting) bias for GSH; Figure S15: Evidence of publication (reporting) bias for GSH-Px; Figure S16: Evidence of publication (reporting) bias for lysozyme; Figure S17: Evidence of publication (reporting) bias for Ig; Figure S18: Evidence of publication (reporting) bias for C3; Figure S19: Evidence of publication (reporting) bias for C4; Figure S20: Evidence of publication (reporting) bias for ACP; Figure S21: Evidence of publication (reporting) bias for AKP; Table S1: Literature sources for growth-related parameters in this meta-analysis; Table S2: Final body weight and weight gain rate of astaxanthin supplemental levels of meta-analysis parameters; Table S3: Specific growth rate and survival rate of astaxanthin supplemental levels of meta-analysis parameters; Table S4: Feed conversion ratio and protein efficiency ratio of astaxanthin supplemental levels of meta-analysis parameters; Table S5: Effect size calculation for lipase and protease comparisons based on random-effect model; Table S6: Effect size calculation for amylase comparisons based on random-effect model; Table S7: Effect size calculation for SOD and CAT comparisons based on random-effect model; Table S8: Effect size calculation for MDA and T-AOC comparisons based on random-effect model; Table S9: Effect size calculation for GSH-Px and GSH comparisons based on random-effect model; Table S10: Effect size calculation for lysozyme and Ig comparisons based on random-effect model; Table S11: Effect size calculation for C3 and C4 comparisons based on random-effect model; Table S12: Effect size calculation for ACP and AKP comparisons based on random-effect model; Table S13: Outcomes of Egger’s regression test to evaluate publication bias during study for outcome indicators. Refs. [13,14,15,16,17,18,19,20,40,41,42,43,48,49,62,63,64,65,66,70,71,79,83,84,85,86,87,88,89,90,91,92,93,94,95,96,97,98,99,100,101,102,103,104,105,106,107,108,109,110,111,112,113,114,115,116,117,118,119,120,121,122,123,124] are cited in the Supplementary Materials.

## Abbreviations

The following abbreviations are used in this manuscript:

FBW	Final body weight
WGR	Weight gain rate
SGR	Specific growth rate
SR	Survival rate
FCR	Feed conversion ratio
PER	Protein efficiency ratio
SOD	Superoxide dismutase
CAT	Catalase
MDA	Malondialdehyde
T-AOC	Total antioxidant capacity
GSH	Glutathione
GSH-Px	Glutathione peroxidase
Ig	Immunoglobulin
ACP	Acid phosphatase
AKP	Alkaline phosphatase
C3	Complement C3
C4	Complement C4
ROS	Reactive oxygen species
