# Supplementary material for: Effectiveness of Astaxanthin as a Feed Supplement to Improve Growth Performance and Feed Utilization in Aquaculture Animals: A Meta-Analysis
Source: Antioxidants (Basel). 2025 May 19;14(5):609. doi: 10.3390/antiox14050609 (PMC12109285; doi:10.3390/antiox14050609)
Supplement: Supplementary file 1 [file antioxidants-14-00609-s001.zip › antioxidants-3594341-supplementary.pdf]

Table S1. Literature sources for growth-related parameters parameters in this meta-analysis

	Species	Habitat	Trophic level	Species category	Growth related parameters					
					FBW	WGR	SGR	FCR	PER	SR
[83]	*Portunus trituberculatus*	Marine	3.34	Shrimp	√	√	NA	NA	NA	NA
[71]	*Portunus trituberculatus*	Marine	3.34	Shrimp	√	√	√	√	√	√
[84]	*Symphysodon spp.*	Freshwater	3.3	Fish	√	√	√	NA	NA	NA
[66]	*Symphysodon spp.*	Freshwater	3.3	Fish	√	√	NA	√	√	NA
[85]	*Symphysodon spp.*	Freshwater	3.3	Fish	√	√	NA	√	√	NA
[86]	*Paramisgurnus dabryanus*	Freshwater	3.3	Fish	√	√	√	√	√	NA
[87]	*Paramisgurnus dabryanus*	Freshwater	3.3	Fish	√	√	NA	√	NA	NA
[88]	*Micropterus salmoides*	Freshwater	3.8	Fish	√	√	√	√	NA	√
[13]	*Micropterus salmoides*	Freshwater	3.8	Fish	√	√	√	√	√	NA
[89]	*Micropterus salmoides*	Freshwater	3.8	Fish	√	√	√	NA	√	√
[90]	*Anguilla rostrata*	Brackish	3.8	Fish	√	√	√	√	NA	√
[91]	*Oncorhynchus mykiss*	Freshwater	4.1	Fish	NA	√	√	√	NA	√
[70]	*Oncorhynchus mykiss*	Freshwater	4.1	Fish	√	√	√	√	√	√
[92]	*Oncorhynchus mykiss*	Freshwater	4.1	Fish	√	√	NA	√	√	NA
[20]	*Oncorhynchus mykiss*	Freshwater	4.1	Fish	√	√	√	√	√	√
[93]	*Oncorhynchus mykiss*	Freshwater	4.1	Fish	√	√	√	√	NA	√
[94]	*Oncorhynchus mykiss*	Freshwater	4.1	Fish	√	√	√	√	NA	√
[14]	*Oncorhynchus mykiss*	Freshwater	4.1	Fish	√	√	√	√	NA	√
[15]	*Oncorhynchus mykiss*	Freshwater	4.1	Fish	√	NA	NA	√	NA	NA
[95]	*Oncorhynchus mykiss*	Freshwater	4.1	Fish	√	NA	NA	√	NA	NA
[96]	*Oncorhynchus mykiss*	Freshwater	4.1	Fish	√	√	√	√	√	NA
[97]	*Epinephelus akaara*	Marine	4.0	Fish	√	√	√	√	NA	√
[98]	*Epinephelus akaara*	Marine	4.0	Fish	√	√	√	√	NA	NA
[16]	*Trachinotus ovatus*	Marine	4.2	Fish	√	√	NA	√	NA	√
[79]	*Trachinotus ovatus*	Marine	4.2	Fish	√	√	NA	√	NA	√
[65]	*Trachinotus ovatus*	Marine	4.2	Fish	√	√	√	√	NA	√
[99]	*Trachinotus ovatus*	Marine	4.2	Fish	NA	√	√	√	NA	√
[49]	*Lates calcarifer*	Marine	3.8	Fish	√	√	√	√	√	NA
[100]	*Pagrus pagrus*	Marine	3.9	Fish	√	NA	√	√	√	NA

Table S1. Literature sources for growth-related parameters parameters in this meta-analysis

	Species	Habitat	Trophic level	Species category	Growth related parameters					
					FBW	WGR	SGR	FCR	PER	SR
[48]	*Pagrus pagrus*	Marine	3.9	Fish	√	√	√	√	NA	NA
[18]	*Cyprinus carpio*	Freshwater	3.1	Fish	NA	√	NA	√	√	NA
[101]	*Pseudosciaena crocea*	Marine	3.7	Fish	√	√	NA	√	√	√
[43]	*Carassius auratus*	Freshwater	2	Fish	√	NA	NA	√	NA	√
[102]	*Takifugu obscurus*	Brackish	3.4	Fish	√	√	√	√	NA	√
[64]	*Eriocheir sinensis*	Freshwater	2.57	Shrimp	√	√	√	√	NA	√
[63]	*Epinephelus lanceolatus*	Marine	4	Fish	√	√	√	√	NA	√
[103]	*Penaeus monodon*	Marine	3.25	Shrimp	√	√	√	√	NA	√
[104]	*Penaeus monodon*	Marine	3.25	Shrimp	√	√	√	√	NA	√
[105]	*Penaeus monodon*	Marine	3.25	Shrimp	√	√	√	NA	NA	√
[106]	*Penaeus monodon*	Marine	3.25	Shrimp	√	√	√	√	NA	√
[62]	*Channa argus*	Freshwater	4.4	Fish	√	√	√	√	NA	NA
[107]	*Channa argus*	Freshwater	4.4	Fish	√	√	√	√	NA	NA
[42]	*Apostichopus japonicas*	Marine	2.1	Sea cucumber	√	√	√	√	NA	√
[108]	*Apostichopus japonicas*	Marine	2.1	Sea cucumber	√	NA	√	NA	NA	NA
[41]	*Marsupenaeus japonicus*	Marine	2.86	Shrimp	√	√	√	NA	NA	√
[109]	*Astronotus ocellatus*	Freshwater	2.8	Fish	NA	√	√	√	√	NA
[110]	*Lophiosilurus alexandri*	Freshwater	4	Fish	√	NA	NA	√	NA	NA
[17]	*Pangasianodon hypophthalmus*	Freshwater	3.1	Fish	√	√	√	√	√	√
[40]	*Plectropomus leopardus*	Marine	4.4	Fish	√	√	√	NA	NA	√
[111]	*O.niloticus×O.mossambicus*	Brackish	4.5	Fish	√	√	√	√	NA	√
[112]	*Pelteobagrus fulvidraco*	Freshwater	3.5	Fish	√	√	√	√	NA	√
[113]	*Pelteobagrus fulvidraco*	Freshwater	3.5	Fish	√	√	NA	√	NA	NA
[114]	*Pelteobagrus fulvidraco*	Freshwater	3.5	Fish	√	√	√	√	√	NA
[19]	*Penaeus vannamei*	Marine	2.54	Shrimp	√	√	√	NA	NA	√
[115]	*Penaeus vannamei*	Marine	2.54	Shrimp	√	√	NA	√	NA	√
[116]	*Penaeus vannamei*	Marine	2.54	Shrimp	√	√	√	√	√	√
[117]	*Salmo labrax*	Marine	3.7	Fish	√	√	√	√	NA	NA
[118]	*Exopalaemon carinicauda*	Marine	3.04	Shrimp	√	√	√	NA	NA	√

Table S1. Literature sources for growth-related parameters parameters in this meta-analysis

	Species	Habitat	Trophic level	Species category	Growth related parameters					
					FBW	WGR	SGR	FCR	PER	SR
[119]	*Larimichthys crocea*	Marine	3.7	Fish	√	√	√	√	NA	NA
[120]	*Cyprinus carpio L.*	Freshwater	3.1	Fish	NA	√	√	NA	√	NA
[121]	*Cyprinus carpio L.*	Freshwater	3.1	Fish	√	√	√	√	NA	√
[122]	*Cyprinus carpio L.*	Freshwater	3.1	Fish	NA	√	√	NA	NA	√
[123]	*Cichlasoma citrinellum XC.synspilum*	Freshwater	3.2	Fish	√	√	√	NA	NA	NA
[124]	*Premnas biaculeatus*	Marine	2.8	Fish	√	NA	√	√	NA	√

Table S2. Final body weight and weight gain rate of astaxanthin supplemental levels of meta-analysis parameters

	Final body weight							Weight gain rate						
Astaxanthin supplemental level	*k*	I^2	Hedges' *g* value	SE	Ci.Lb	Ci.Ub	*P*-value	*k*	I^2	Hedges' *g* value	SE	Ci.Lb	Ci.Ub	*P*-value
0-100	87	72.64	1.6162	0.2103	1.204	2.0283	<0.0001	75	53.90	1.6298	0.1707	1.2952	1.9644	<0.0001
100-200	56	71.62	1.5502	0.2578	1.0448	2.0556	<0.0001	55	74.16	2.0684	0.2897	1.5007	2.6361	<0.0001
200-300	25	67.46	1.6160	0.3644	0.9017	2.3303	<0.0001	24	47.43	1.8568	0.3031	1.2627	2.4509	<0.0001
300-400	12	83.85	2.9326	0.8852	1.1976	4.6676	0.0009	12	91.09	3.1163	1.2733	0.6207	5.6118	0.0144
400-500	10	78.83	0.5140	0.6731	-0.8053	1.8333	0.4451	9	94.43	1.5334	1.6848	-1.7687	4.8355	0.3628
500-1000	13	57.42	2.3713	0.4758	1.4387	3.3039	<0.0001	16	74.01	2.4917	0.5624	1.3894	3.5941	<0.0001
≥1000	15	75.14	1.9146	0.5506	0.8355	2.9937	0.0005	20	77.13	1.8671	0.5086	0.8703	2.8640	0.0002

k, sample size; I^2 the percentage changes resulting from heterogeneity among different studies; SE, standard error; Ci.Lb, confidence interval lower bound; Ci.Ub, confidence interval upper bound.

Table S3. Specific growth rate and survival rate of astaxanthin supplemental levels of meta-analysis parameters

	Specific growth rate							Survival rate						
Astaxanthin supplemental level	*k*	I^2	Hedges' *g* value	SE	Ci.Lb	Ci.Ub	*P*-value	*k*	I^2	Hedges' *g* value	SE	Ci.Lb	Ci.Ub	*P*-value
0-100	66	72.44	1.7215	0.2427	1.2458	2.1973	<0.0001	56	0	0.2914	0.1128	0.0703	0.5126	0.0098
100-200	46	77.81	1.905	0.3349	1.2486	2.5614	<0.0001	26	60.74	0.6843	0.2831	0.1295	1.2391	0.0156
200-300	21	68.58	1.3002	0.3794	0.5566	2.0438	0.0006	12	65.41	0.2091	0.4930	-0.7571	1.1754	0.6714
300-400	8	81.77	1.6106	0.8877	-0.1294	3.3505	0.0696	3	0	-0.1098	0.4725	-1.0359	0.8162	0.8162
400-500	7	50.84	0.1602	0.4873	-0.7948	1.1153	0.7423	8	41.76	1.2190	0.4831	0.2721	2.1660	0.0116
500-1000	13	57.56	1.3843	0.4113	0.5782	2.1904	0.0008	11	0	0.8474	0.2687	0.3207	1.3741	0.0016
≥1000	16	75.17	1.5554	0.5318	0.5132	2.5977	0.0034	8	57.35	0.7538	0.5090	-0.2437	1.7514	0.1386

k, sample size; I^2 the percentage changes resulting from heterogeneity among different studies; SE, standard error; Ci.Lb, confidence interval lower bound; Ci.Ub, confidence interval upper bound.

Table S4. Feed conversion ratio and protein efficiency ratio of astaxanthin supplemental levels of meta-analysis parameters

	Feed conversion ratio							Protein efficiency ratio						
Astaxanthin supplemental level	*k*	I^2	Hedges' *g* value	SE	Ci.Lb	Ci.Ub	*P*-value	*k*	I^2	Hedges' *g* value	SE	Ci.Lb	Ci.Ub	*P*-value
0-100	71	54.90	-0.8652	0.1606	-1.1800	-0.5505	<0.0001	24	49.72	0.9311	0.2800	0.3822	1.4799	0.0009
100-200	47	69.61	-1.0607	0.2511	-1.5529	-0.5685	<0.0001	16	70.88	1.4322	0.4786	0.4942	2.3703	0.0028
200-300	20	43.71	-0.6554	0.271	-1.1866	-0.1242	0.0156	5	0	1.5168	0.4234	0.6869	2.3466	0.0003
300-400	10	78.78	-0.8493	0.7211	-2.2627	0.5641	0.2389	1	0	-0.8405	0.8518	-2.5099	0.8290	0.3238
400-500	6	95.53	-1.6521	2.5018	-6.5556	3.2514	0.5090	1	0	0.5980	0.8345	-1.0376	2.2337	0.4736
500-1000	14	84.64	-1.7835	0.7030	-3.1614	-0.4056	0.0112	3	0	-0.4513	0.4805	-1.3931	0.4905	0.3476
≥1000	12	84.18	-1.564	0.7600	-3.0536	-0.0744	0.0396	4	79.50	3.9150	1.6510	0.6791	7.1509	0.0177

k, sample size; I^2 the percentage changes resulting from heterogeneity among different studies; SE, standard error; Ci.Lb, confidence interval lower bound; Ci.Ub, confidence interval upper bound.

Table S5. Effect size calculation for lipase and protease comparisons based on random-effect model

	Lipase							Protease						
	k	I^2	Hedges' *g* value	SE	Ci.Lb	Ci.Ub	*P*-value	*k*	I^2	Hedges' *g* value	SE	Ci.Lb	Ci.Ub	*P*-value
All species	14	88.14	10.4932	2.0013	6.5707	14.4158	<0.0001	14	56.11	4.8854	0.6784	3.5558	6.2151	<0.0001
Fish	10	78.87	8.3388	1.6130	5.1773	11.5003	<0.0001	10	50.73	5.6455	0.8503	3.9789	7.3121	<0.0001
Crustacean	1	0	3.9894	1.4117	1.2225	6.7563	0.0047	1	0	1.6597	0.9467	-0.1958	3.5152	0.0796
Sea cucumber	3	46.23	36.5837	8.9732	18.9966	54.1708	<0.0001	3	0	4.0148	0.8507	2.3475	5.6821	<0.0001
Marine species	7	98.35	15.1302	6.3639	2.6571	27.6032	0.0174	7	20.88	3.2364	0.5640	2.1310	4.3418	<0.0001
Freshwater species	7	57.44	10.7707	1.8893	7.0678	14.4736	<0.0001	7	0	6.4566	0.7995	4.8895	8.0236	<0.0001
low trophic level	7	93.16	17.9373	5.3471	7.4572	28.4174	0.0008	7	65.43	4.8502	1.0644	2.7640	6.9365	<0.0001
middle trophic level	4	57.10	11.0241	2.6182	5.8926	16.1556	<0.0001	4	0	6.2356	1.0245	4.2275	8.2436	<0.0001
high trophic level	3	67.82	3.9337	1.5292	0.9365	6.9310	<0.0001	3	0	3.2848	0.7649	1.7856	4.7840	<0.0001

k, sample size; I^2 the percentage changes resulting from heterogeneity among different studies; SE, standard error; Ci.Lb, confidence interval lower bound; Ci.Ub, confidence interval upper bound.

Table S6. Effect size calculation for amylase comparisons based on random-effect model

	Amylase						
	k	I^2	Hedges' *g* value	SE	Ci.Lb	Ci.Ub	*P*-value
All species	14	85.21	5.7277	1.1803	3.4143	8.0411	<0.0001
Fish	10	73.29	4.2001	0.9772	2.2848	6.1154	<0.0001
Crustacean	1	0	2.5931	1.1077	0.4221	4.7642	0.0192
Sea cucumber	3	0	10.3532	1.8184	6.7892	13.9171	<0.0001
Marine species	7	76.70	6.1096	1.4666	3.2350	8.9841	<0.0001
Freshwater species	7	96.08	7.2818	3.0445	1.3148	13.2489	0.0168
low trophic level	7	81.69	4.9494	1.3928	2.2195	7.6793	0.0004
middle trophic level	4	94.44	15.9406	7.7833	0.6857	31.1955	0.0406
high trophic level	3	58.97	4.2862	1.4247	1.4939	7.0785	0.0026

k, sample size; I^2 the percentage changes resulting from heterogeneity among different studies; SE, standard error; Ci.Lb, confidence interval lower bound; Ci.Ub, confidence interval upper bound.

Table S7. Effect size calculation for SOD and CAT comparisons based on random-effect model

	SOD							CAT						
	k	I^2	Hedges' *g* value	SE	Ci.Lb	Ci.Ub	*P*-value	*k*	I^2	Hedges' *g* value	SE	Ci.Lb	Ci.Ub	*P*-value
All species	80	88.79	0.1138	0.3567	-0.5854	0.813	0.7497	57	85.89	-0.2970	0.3820	-1.0457	0.4517	0.4368
Fish	48	92.06	1.4216	0.6018	0.2421	2.6011	0.0182	29	89.88	1.3806	0.7308	-0.0519	2.8130	0.0589
Crustacean	32	45.94	-1.2879	0.2261	-1.7310	-0.8448	<0.0001	28	53.11	-1.4392	0.2645	-1.9577	-0.9208	<0.0001
Marine species	48	77.79	-1.1977	0.3243	-1.8332	-0.5622	<0.0001	41	80.74	-1.1305	0.3737	-1.8630	-0.3980	0.0025
Freshwater species	32	85.52	2.0899	0.5162	1.0782	3.1017	<0.0001	16	86.79	2.2474	0.8273	0.6259	3.8690	0.0066
low trophic level	7	83.80	-0.1493	1.0005	-2.1103	1.8116	0.8813	3	0	2.4070	0.6262	1.1797	3.6343	0.0001
middle trophic level	38	83.44	-1.3860	0.4227	-2.2144	-0.5576	0.0010	38	72.23	-1.5128	0.3336	-2.1666	-0.8590	<0.0001
high trophic level	35	87.60	1.8898	0.5260	0.8590	2.9207	0.0003	16	82.94	1.8989	0.6742	0.5775	3.2204	0.0049

k, sample size; I^2 the percentage changes resulting from heterogeneity among different studies; SE, standard error; Ci.Lb, confidence interval lower bound; Ci.Ub, confidence interval upper bound.

Table S8. Effect size calculation for MDA and T-AOC comparisons based on random-effect model

	MDA							T-AOC						
	k	I^2	Hedges' *g* value	SE	Ci.Lb	Ci.Ub	*P*-value	*k*	I^2	Hedges' *g* value	SE	Ci.Lb	Ci.Ub	*P*-value
All species	55	85.14	-3.8318	0.4599	-4.7333	-2.9304	<0.0001	44	79.10	3.4295	0.4117	2.6227	4.2364	<0.0001
Fish	44	89.70	-4.5072	0.6330	-5.7478	-3.2665	<0.0001	35	70.22	3.8013	0.4267	2.9650	4.6376	<0.0001
Crustacean	11	16.69	-2.5861	0.3774	-3.3257	-1.8465	<0.0001	9	84.94	1.7375	0.8164	0.1374	3.3375	0.0333
Marine species	29	81.10	-2.7542	0.5253	-3.7837	-1.7248	<0.0001	21	68.29	1.9795	0.4014	1.1928	2.7662	<0.0001
Freshwater species	26	82.82	-4.8188	0.6904	-6.1719	-3.4657	<0.0001	23	78.74	5.2069	0.7099	3.8155	6.5983	<0.0001
low trophic level	4	37.84	-1.9113	0.6536	-3.1923	-0.6303	0.0035	4	0	4.5216	0.7914	2.9705	6.0728	<0.0001
middle trophic level	17	86.65	-7.2386	1.2712	-9.7302	-4.7470	<0.0001	10	97.53	5.2927	2.1989	0.9829	9.6025	<0.0001
high trophic level	34	85.91	-3.1206	0.5376	-4.1743	-2.0669	<0.0001	30	58.56	3.1992	0.3575	2.4985	3.8999	<0.0001

k, sample size; I^2 the percentage changes resulting from heterogeneity among different studies; SE, standard error; Ci.Lb, confidence interval lower bound; Ci.Ub, confidence interval upper bound.

Table S9. Effect size calculation for GSH-Px and GSH comparisons based on random-effect model

	GSH-Px							GSH						
	k	I^2	Hedges' *g* value	SE	Ci.Lb	Ci.Ub	*P*-value	*k*	I^2	Hedges' *g* value	SE	Ci.Lb	Ci.Ub	*P*-value
All species	36	84.71	2.5790	0.4863	1.6259	3.5321	<0.0001	24	95.06	1.5176	1.3342	-1.0974	4.1327	0.2553
Fish	27	81.02	3.5555	0.5900	2.3991	4.7120	<0.0001	12	87.76	6.6119	1.4734	3.7241	9.4997	<0.0001
Crustacean	9	54.81	0.2226	0.4034	-0.5680	1.0132	0.5810	12	80.60	-3.3174	0.8518	-4.9868	-1.6479	<0.0001
Marine species	12	81.44	1.9780	0.6937	0.6183	3.3377	0.0044	14	85.99	-2.5627	0.8800	-4.2875	-0.8380	0.0036
Freshwater species	24	86.59	2.9754	0.6751	1.6521	4.2986	<0.0001	10	83.82	8.0209	1.7161	4.6574	11.3845	<0.0001
Low trophic level	4	0	0.4650	0.423	-0.3641	1.2940	0.2717	NA	NA	NA	NA	NA	NA	NA
Middle trophic level	12	94.42	3.7709	1.3270	1.1700	6.3717	0.0045	17	96.45	1.0394	2.0021	-2.8845	4.9634	0.6036
High trophic level	20	77.78	2.7767	0.6045	1.5920	3.9614	<0.0001	7	32.43	3.1139	0.5845	1.9682	4.2595	<0.0001

k, sample size; I^2 the percentage changes resulting from heterogeneity among different studies; SE, standard error; Ci.Lb, confidence interval lower bound; Ci.Ub, confidence interval upper bound; NA, not applicable.

Table S10. Effect size calculation for lysozyme and Ig comparisons based on random-effect model

	Lysozyme							Ig						
	k	I^2	Hedges' *g* value	SE	Ci.Lb	Ci.Ub	*P*-value	*k*	I^2	Hedges' *g* value	SE	Ci.Lb	Ci.Ub	*P*-value
All species	46	88.20	2.8336	0.5042	1.8454	3.8218	<0.0001	17	88.64	2.4234	0.9256	0.6093	4.2376	0.0088
Fish	39	82.63	2.7167	0.4481	1.8383	3.5950	<0.0001	17	88.64	2.4234	0.9256	0.6093	4.2376	0.0088
Crustacean	4	0	-1.1277	0.4412	-1.9924	-0.2630	0.0106	NA	NA	NA	NA	NA	NA	NA
Sea cucumber	3	37.04	12.9464	2.9289	7.2060	18.6869	<0.0001	NA	NA	NA	NA	NA	NA	NA
Marine species	17	88.90	6.0037	1.2351	3.5828	8.4245	<0.0001	9	88.08	0.2086	1.0525	-1.8544	2.2715	0.8429
Freshwater species	29	76.43	1.4112	0.3864	0.6539	2.1685	0.0003	5	49.01	4.8906	1.0661	2.8010	6.9801	<0.0001
Low trophic level	12	93.10	2.9508	1.3339	0.3364	5.5652	0.0270	3	75.26	5.5965	2.1941	1.2960	9.8969	0.0108
Middle trophic level	16	86.33	4.1819	0.8812	2.4548	5.9090	<0.0001	5	0	1.6865	0.4335	0.8369	2.5360	<0.0001
High trophic level	18	85.09	1.9470	0.6638	0.6459	3.2481	0.0034	12	91.58	2.8042	1.4855	-0.1072	5.7156	0.0591

k, sample size; I^2 the percentage changes resulting from heterogeneity among different studies; SE, standard error; Ci.Lb, confidence interval lower bound; Ci.Ub, confidence interval upper bound; NA, not applicable.

Table S11. Effect size calculation for C3 and C4 comparisons based on random-effect model

	C3							C4						
	k	I^2	Hedges' *g* value	SE	Ci.Lb	Ci.Ub	*P*-value	*k*	I^2	Hedges' *g* value	SE	Ci.Lb	Ci.Ub	*P*-value
All species	11	86.06	2.3005	0.8665	0.6023	3.9988	0.0079	9	0	0.7601	0.2584	0.2537	1.2666	0.0033
Fish	11	86.06	2.3005	0.8665	0.6023	3.9988	0.0079	9	0	0.7601	0.2584	0.2537	1.2666	0.0033
Marine species	3	91.11	-1.4437	2.1807	-5.7178	2.8304	0.5080	3	92.52	-0.9354	2.4395	-5.7168	3.8460	0.7014
Freshwater species	6	0	2.6804	0.3823	1.9310	3.4297	<0.0001	6	0	0.7872	0.2851	0.2284	1.3459	0.0058
Brackish species	2	0	5.8537	1.1558	3.5884	8.1189	<0.0001							
Low trophic level	3	16.07	3.1204	0.7921	1.5680	4.6728	<0.0001	3	0	1.1912	0.5145	0.1828	2.1997	0.0206
Middle trophic level	5	58.54	3.4233	0.7095	2.0327	4.8140	<0.0001	3	0	0.6081	0.3425	-0.0631	1.2794	0.0758
High trophic level	3	91.11	-1.4437	2.1807	-5.7178	2.8304	0.5080	3	92.52	-0.9354	2.4395	-5.7168	3.8460	0.7014

k, sample size; I^2 the percentage changes resulting from heterogeneity among different studies; SE, standard error; Ci.Lb, confidence interval lower bound; Ci.Ub, confidence interval upper bound; NA, not applicable.

Table S12. Effect size calculation for ACP and AKP comparisons based on random-effect model

	ACP							AKP						
	k	I^2	Hedges' *g* value	SE	Ci.Lb	Ci.Ub	*P*-value	*k*	I^2	Hedges' *g* value	SE	Ci.Lb	Ci.Ub	*P*-value
All species	24	89.93	3.4594	0.6896	2.1078	4.8109	$<$0.0001	27	86.35	2.365	0.5469	1.2930	3.4369	$<$0.0001
Fish	12	95.33	4.425	1.3645	1.7506	7.0994	0.0012	15	88.65	2.7924	0.8345	1.1569	4.4279	0.0008
Crustacean	9	72.17	2.0531	0.6269	0.8244	3.2819	0.0011	9	68.30	0.9157	0.5120	-0.0879	1.9192	0.0737
Sea cucumber	3	46.89	6.6176	1.7421	3.2031	10.0321	0.0001	3	60.32	7.5981	2.2842	3.1212	12.0749	0.0009
Marine species	11	87.17	5.6181	1.2592	3.1502	8.0861	$<$0.0001	11	86.33	2.6776	0.9404	0.8345	4.5207	0.0044
Freshwater species	11	82.93	1.9061	0.6778	0.5777	3.2345	0.0049	14	87.63	1.9056	0.7475	0.4405	3.3707	0.0108
Brackish species	2	0	1.5057	0.5669	0.3947	2.6167	0.0079	2	0	4.0059	0.8699	2.3009	5.7109	$<$0.0001
Low trophic level	10	83.70	4.5599	1.1464	2.3131	6.8067	$<$0.0001	10	94.86	5.1218	1.8772	1.4426	8.8010	0.0064
Middle trophic level	10	65.40	1.4781	0.4306	0.634	2.3221	0.0006	14	77.52	1.9803	0.5091	0.9825	2.9780	0.0001
High trophic level	4	84.83	8.2784	3.3757	1.6622	14.8946	0.0142	3	88.32	0.7504	1.9372	-3.0464	4.5472	0.6985

k, sample size; I^2 the percentage changes resulting from heterogeneity among different studies; SE, standard error; Ci.Lb, confidence interval lower bound; Ci.Ub, confidence interval upper bound; NA, not applicable.

Table S13: Outcomes of Egger's regression test to evaluate publication bias during the study for outcome indicators.

**Outcome indicator**	Egger's regression test	
	z value	p-value
FBW	17.8481	< 0.0001
WGR	17.6163	< 0.0001
SGR	16.1141	< 0.0001
FCR	-10.6735	< 0.0001
PER	9.0891	< 0.0001
SR	9.1725	< 0.0001
SOD	2.9343	0.0033
CAT	4.6854	< 0.0001
MDA	-14.9391	< 0.0001
LZM	10.8380	< 0.0001
GSH	4.8751	< 0.0001
GSH-Px	8.3417	< 0.0001
Ig	0.9844	0.3249
C4	-2.1028	0.0355
C3	-0.0699	0.9443
T-AOC	12.6192	< 0.0001
ACP	9.6998	< 0.0001
AKP	8.2416	< 0.0001
Amylase	7.1560	< 0.0001
Protease	5.3925	< 0.0001
Lipase	8.1435	< 0.0001

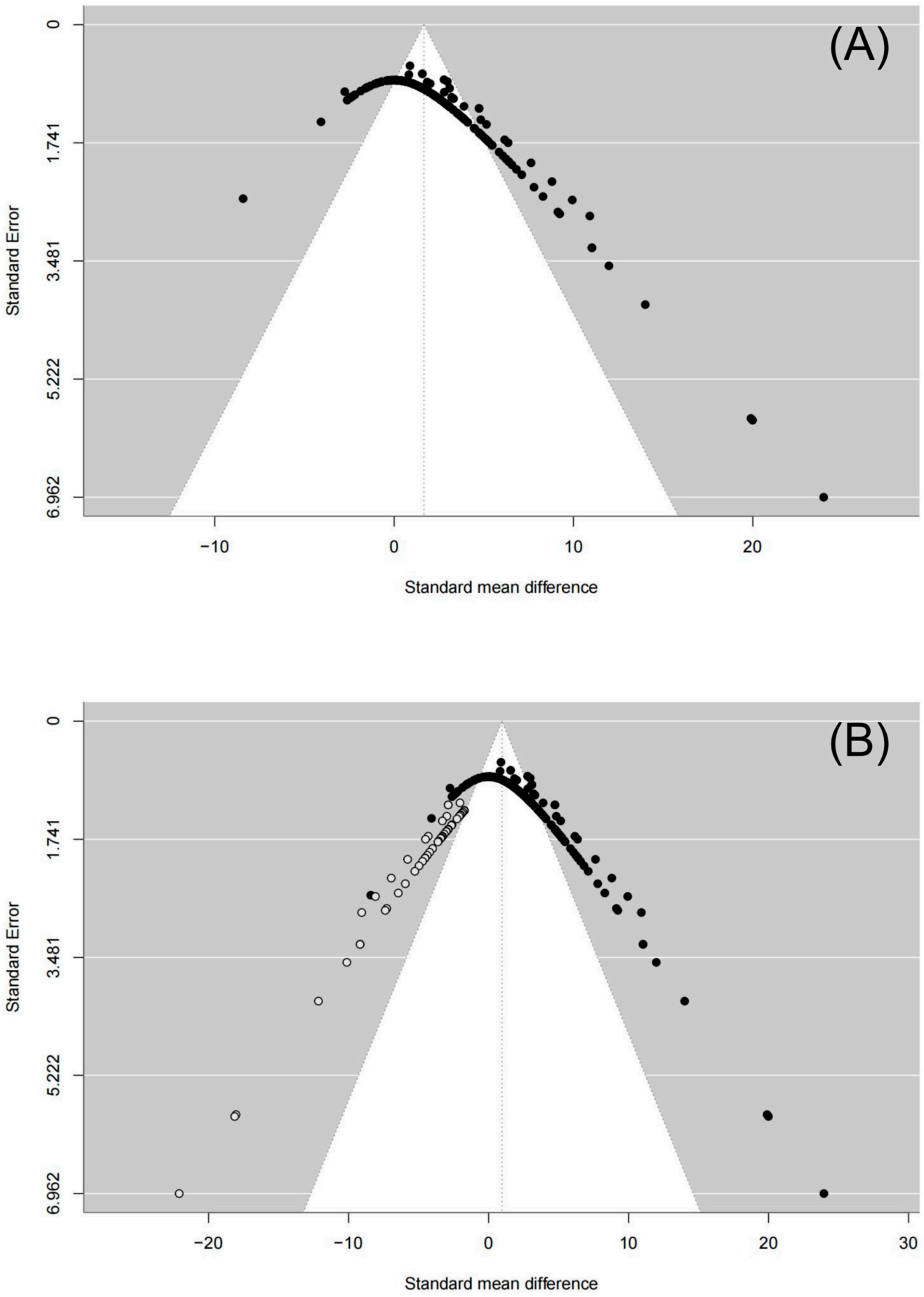

Figure S1. Evidence of publication (reporting) bias. (a) Funnel plot of standardized mean difference of final body weight (FBW); in the absence of bias, these points should be similar to a symmetrical inverted funnel shape. (b) Display the funnel plot of the missing studies supplemented by the 'trim and fill' method (shown in white); the white vertical line indicates the possible summary results if the theoretical missing studies are taken into account.

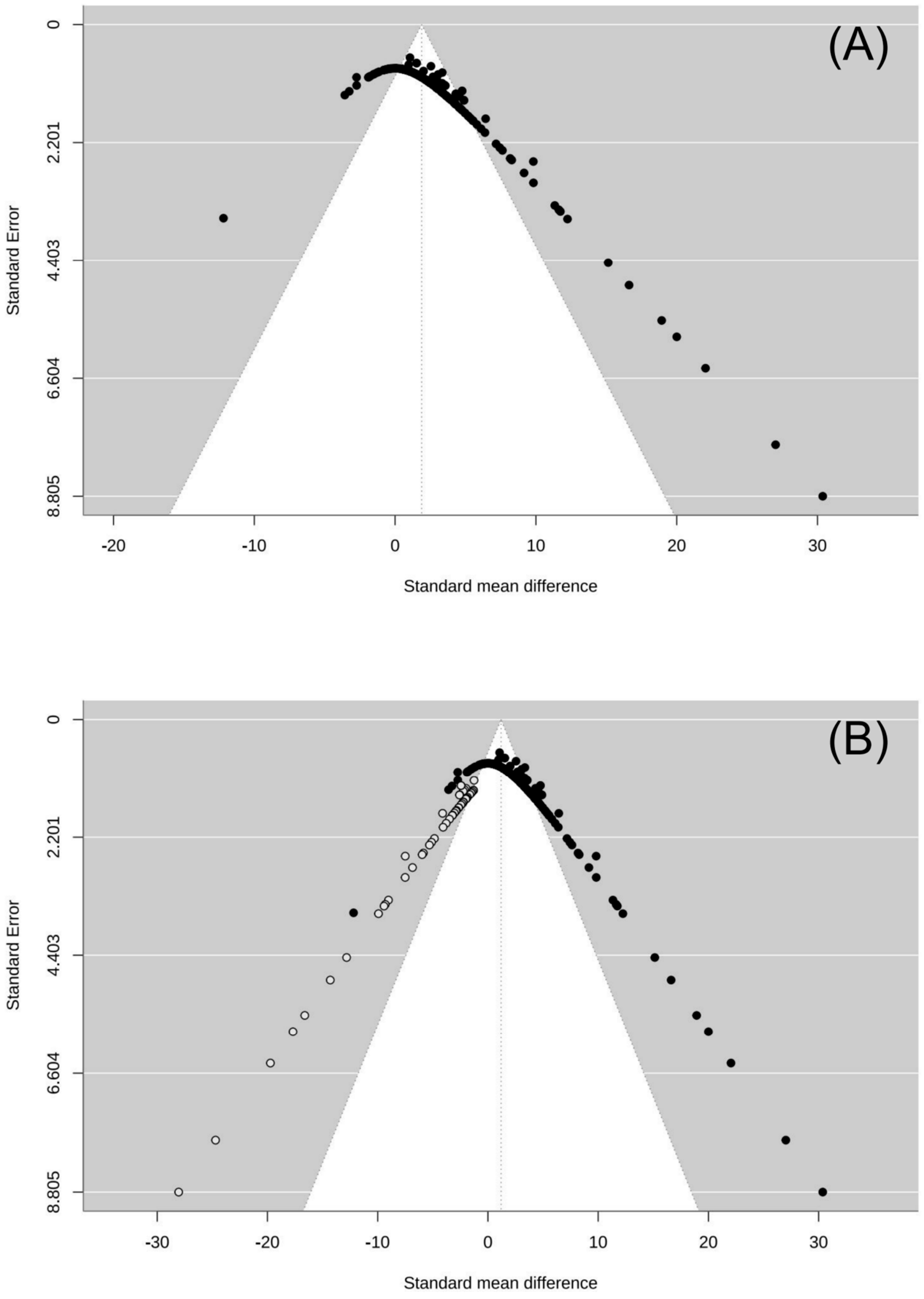

Figure S2. Evidence of publication (reporting) bias for weight gain rate (WGR). (a) Funnel plot of standardized mean difference; in the absence of bias, these points should be similar to a symmetrical inverted funnel shape. (b) Display the funnel plot of the missing studies supplemented by the 'trim and fill' method (shown in white); the white vertical line indicates the possible summary results if the theoretical missing studies are taken into account.

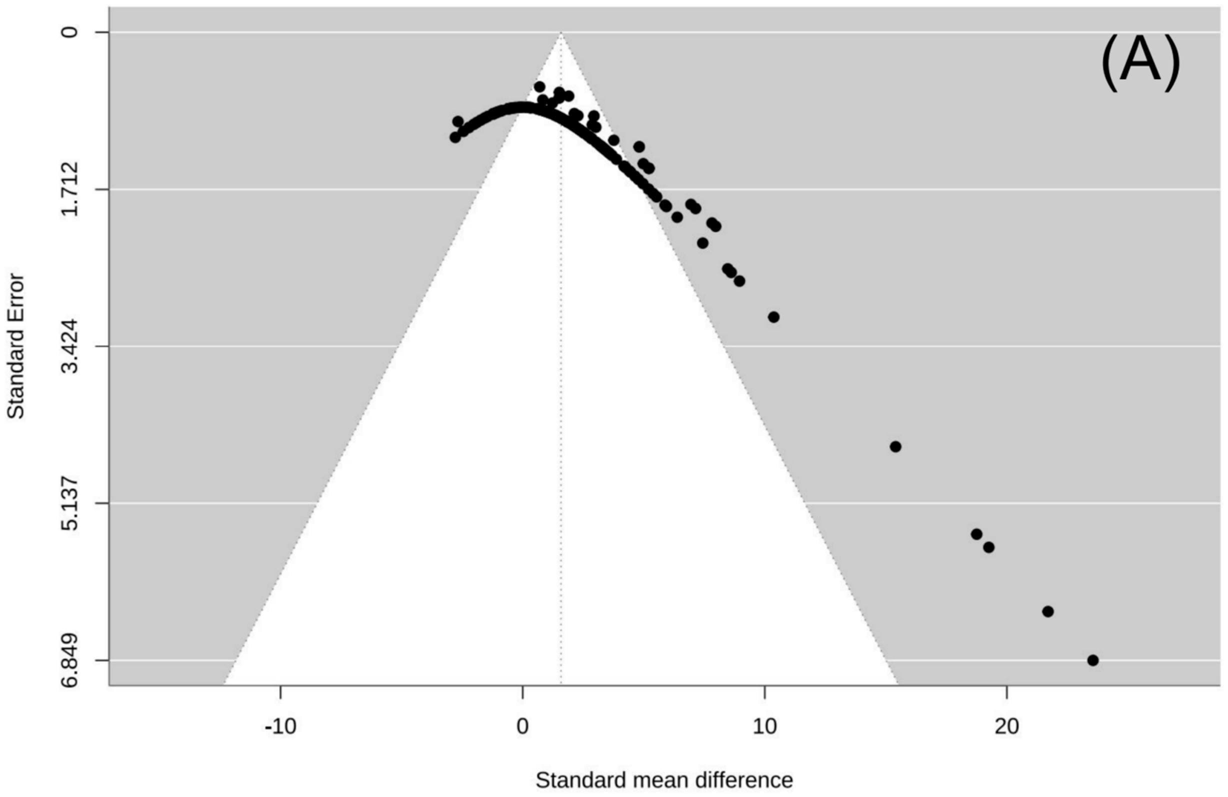

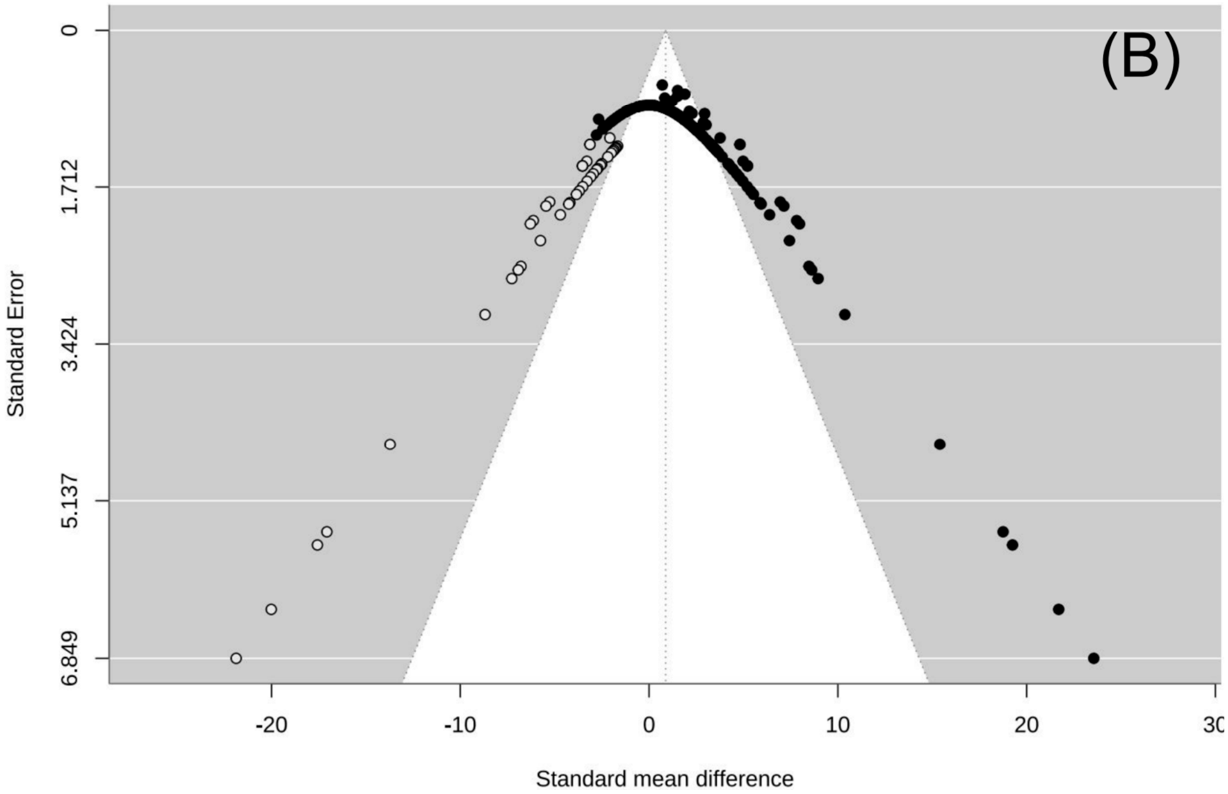

Figure S3. Evidence of publication (reporting) bias for specific growth ratio (SGR). (a) Funnel plot of standardized mean difference; in the absence of bias, these points should be similar to a symmetrical inverted funnel shape. (b) Display the funnel plot of the missing studies supplemented by the 'trim and fill' method (shown in white); the white vertical line indicates the possible summary results if the theoretical missing studies are taken into account.

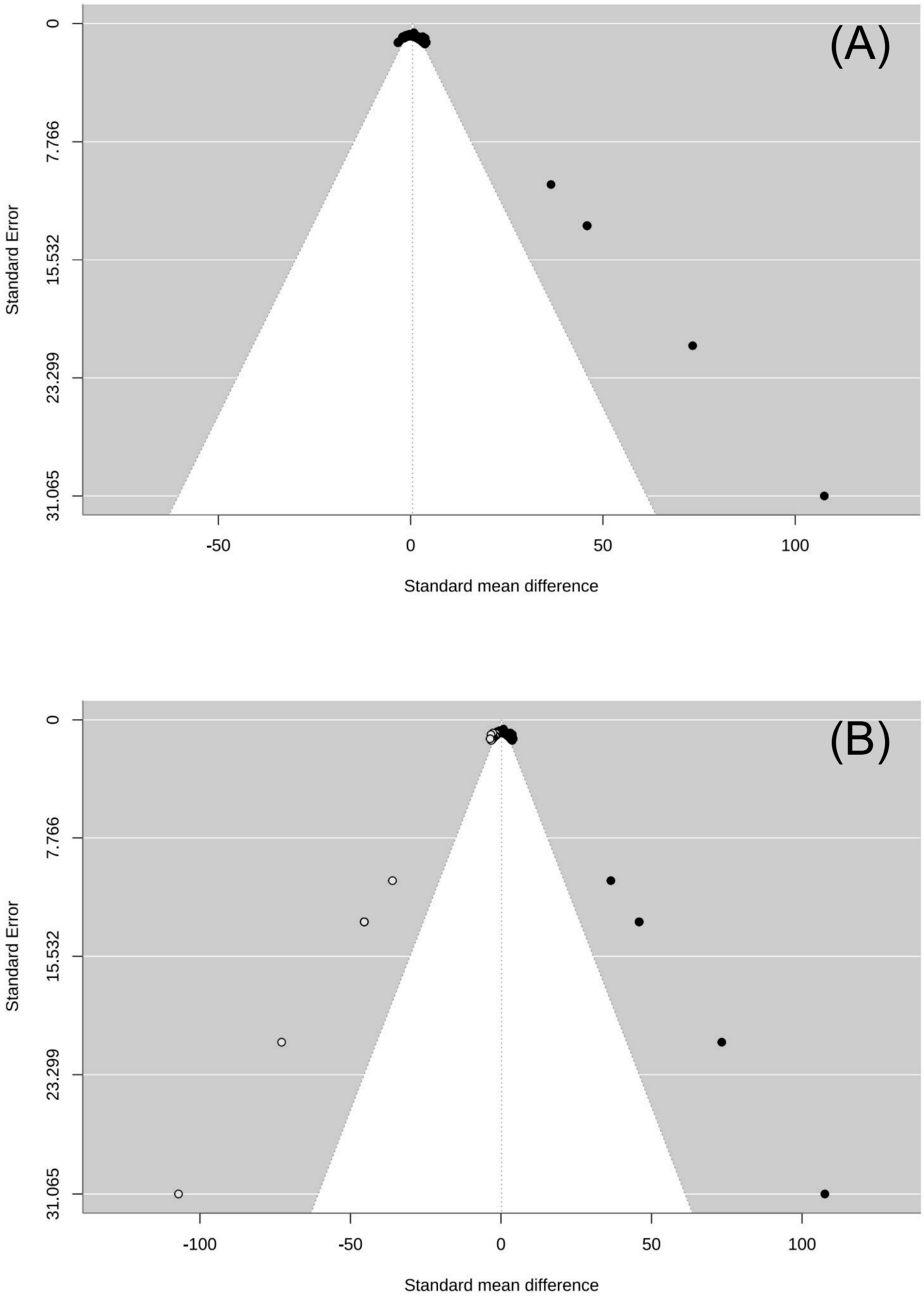

Figure S4. Evidence of publication (reporting) bias for survival rate (SR). (a) Funnel plot of standardized mean difference; in the absence of bias, these points should be similar to a symmetrical inverted funnel shape. (b) Display the funnel plot of the missing studies supplemented by the 'trim and fill' method (shown in white); the white vertical line indicates the possible summary results if the theoretical missing studies are taken into account.

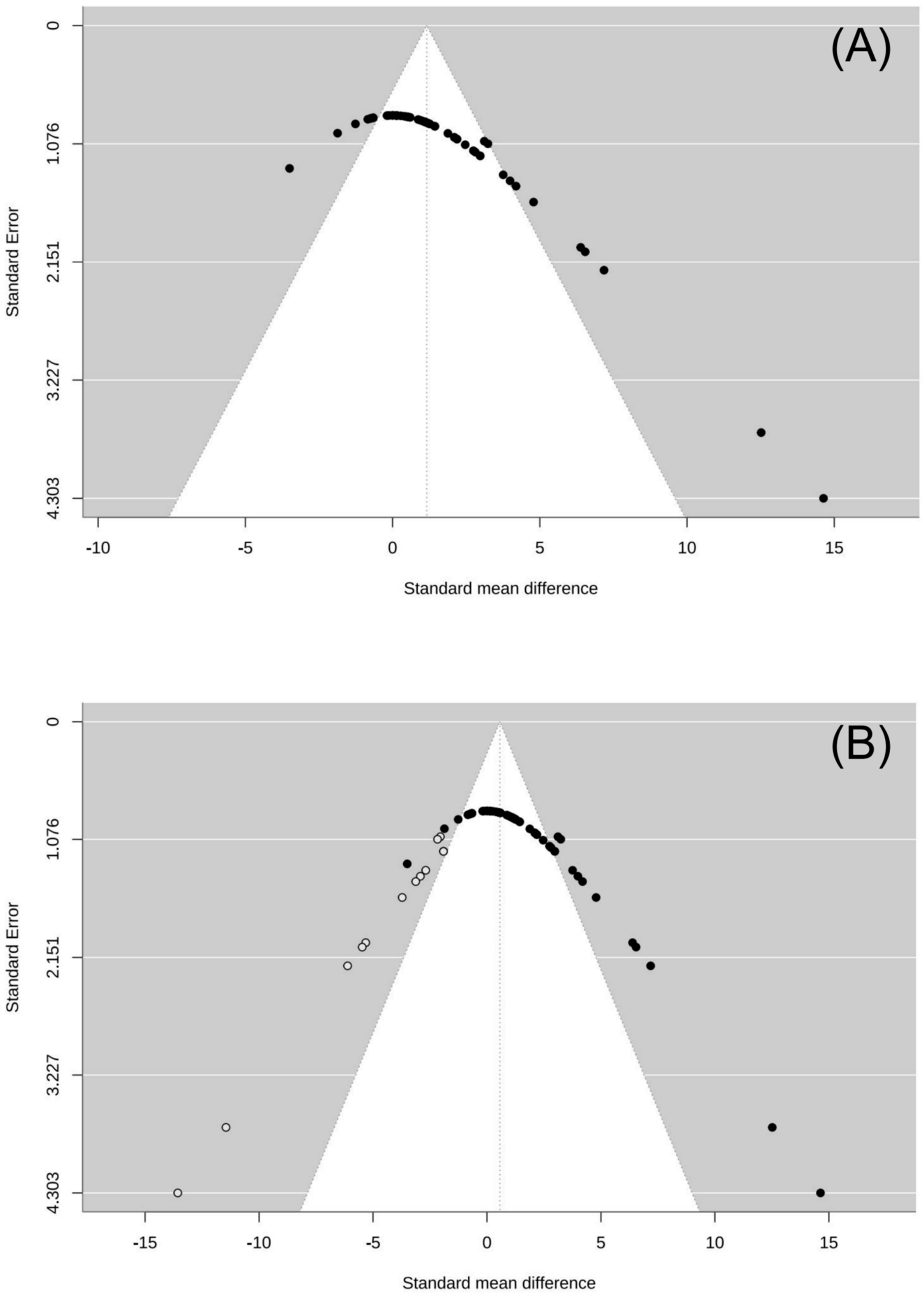

Figure S5. Evidence of publication (reporting) bias for protein efficiency ratio (PER). (a) Funnel plot of standardized mean difference; in the absence of bias, these points should be similar to a symmetrical inverted funnel shape. (b) Display the funnel plot of the missing studies supplemented by the 'trim and fill' method (shown in white); the white vertical line indicates the possible summary results if the theoretical missing studies are taken into account.

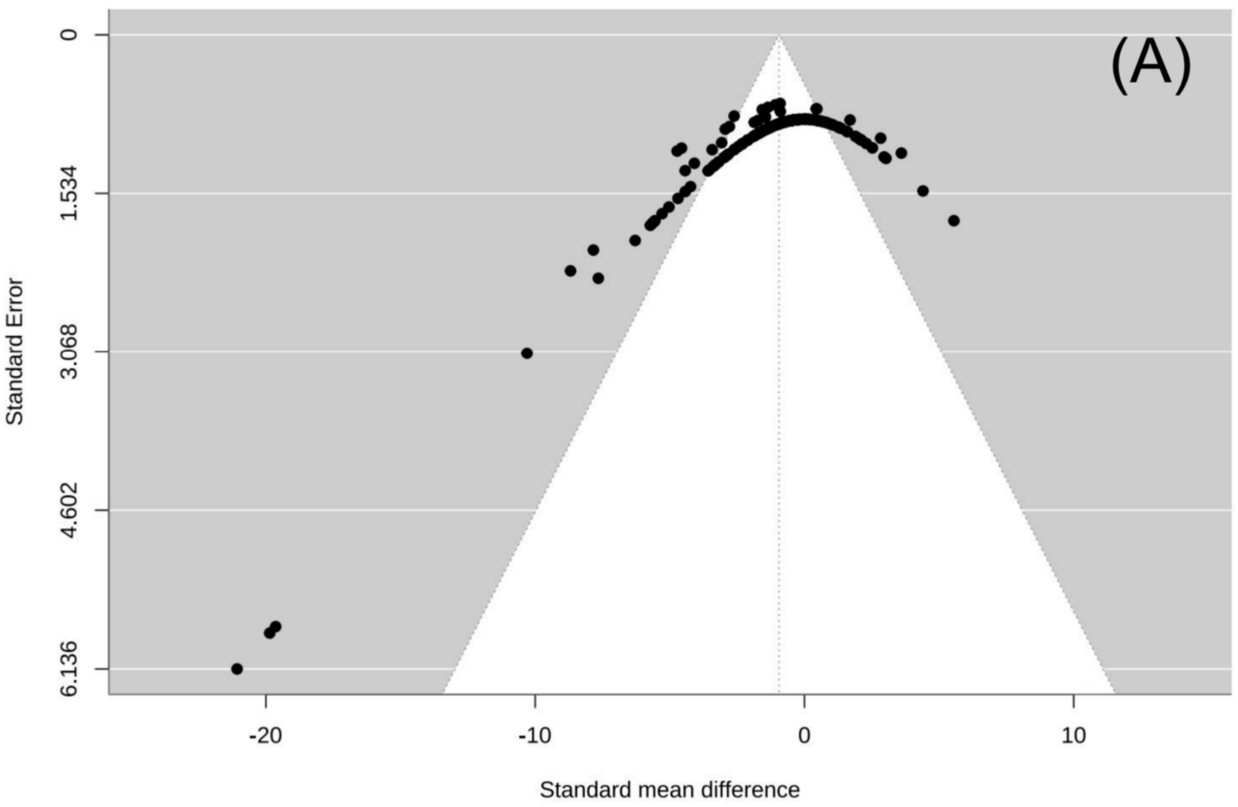

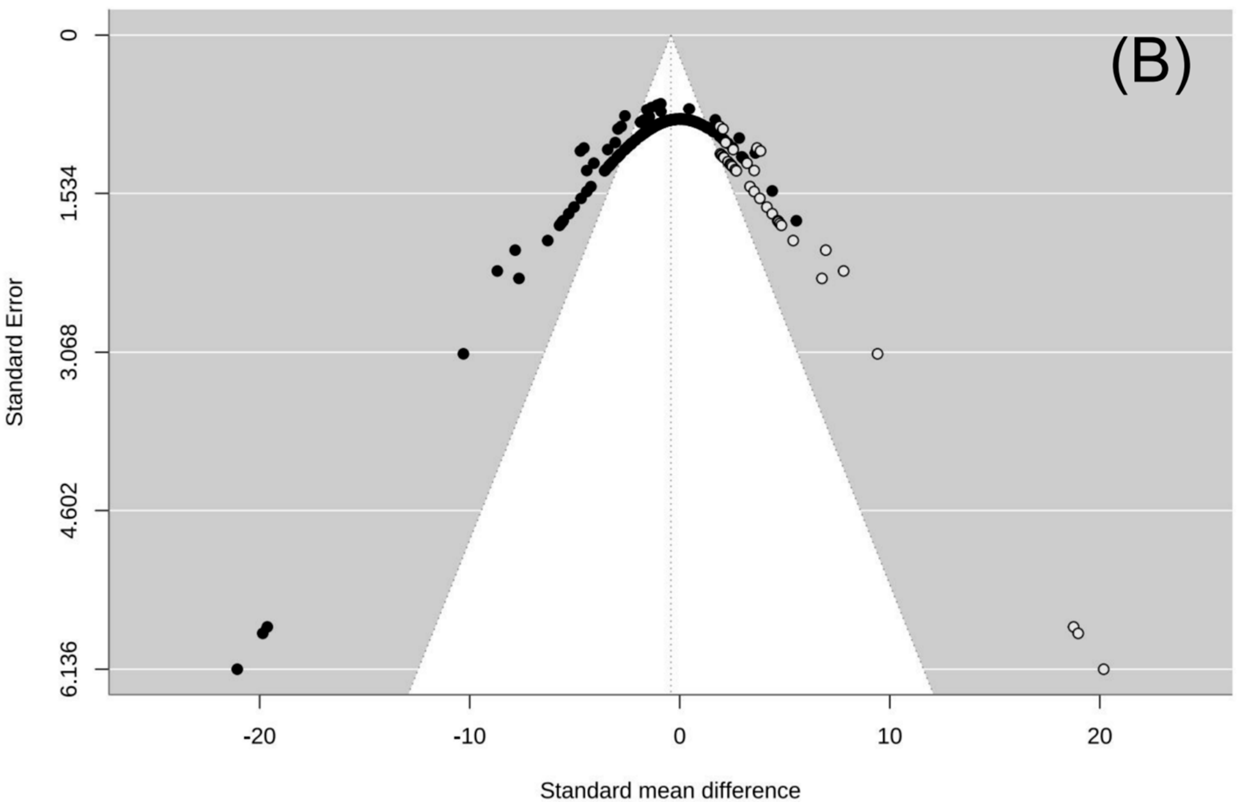

Figure S6. Evidence of publication (reporting) bias for feed conversion ratio (FCR). (a) Funnel plot of standardized mean difference; in the absence of bias, these points should be similar to a symmetrical inverted funnel shape. (b) Display the funnel plot of the missing studies supplemented by the 'trim and fill' method (shown in white); the white vertical line indicates the possible summary results if the theoretical missing studies are taken into account.

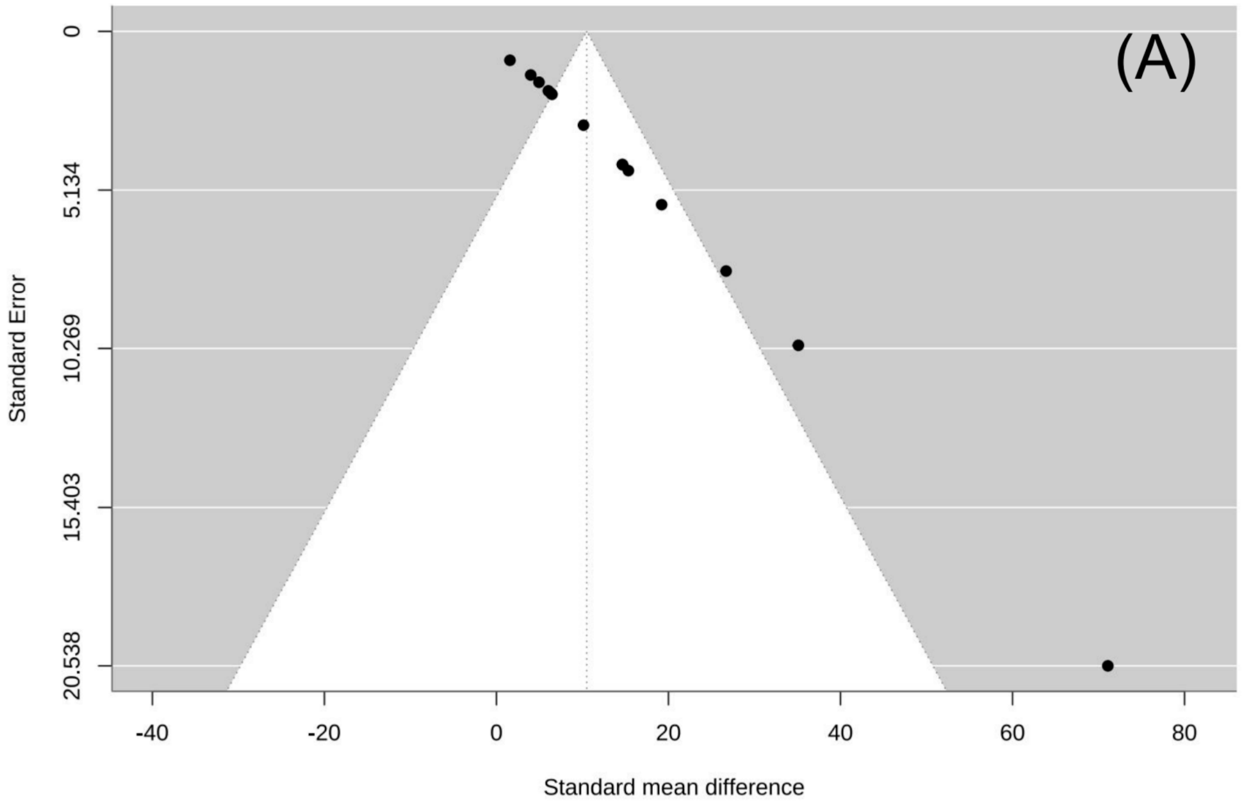

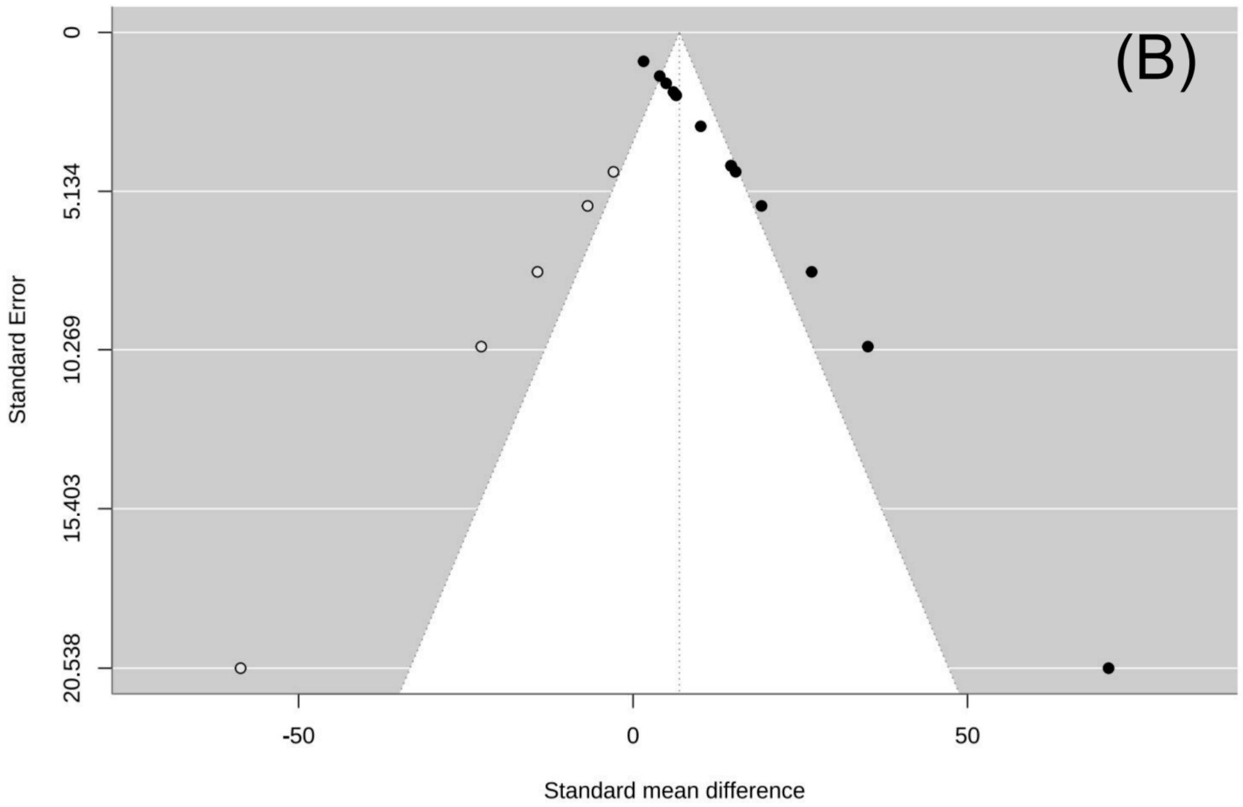

Figure S7. Evidence of publication (reporting) bias for lipase. (a) Funnel plot of standardized mean difference; in the absence of bias, these points should be similar to a symmetrical inverted funnel shape. (b) Display the funnel plot of the missing studies supplemented by the 'trim and fill' method (shown in white); the white vertical line indicates the possible summary results if the theoretical missing studies are taken into account.

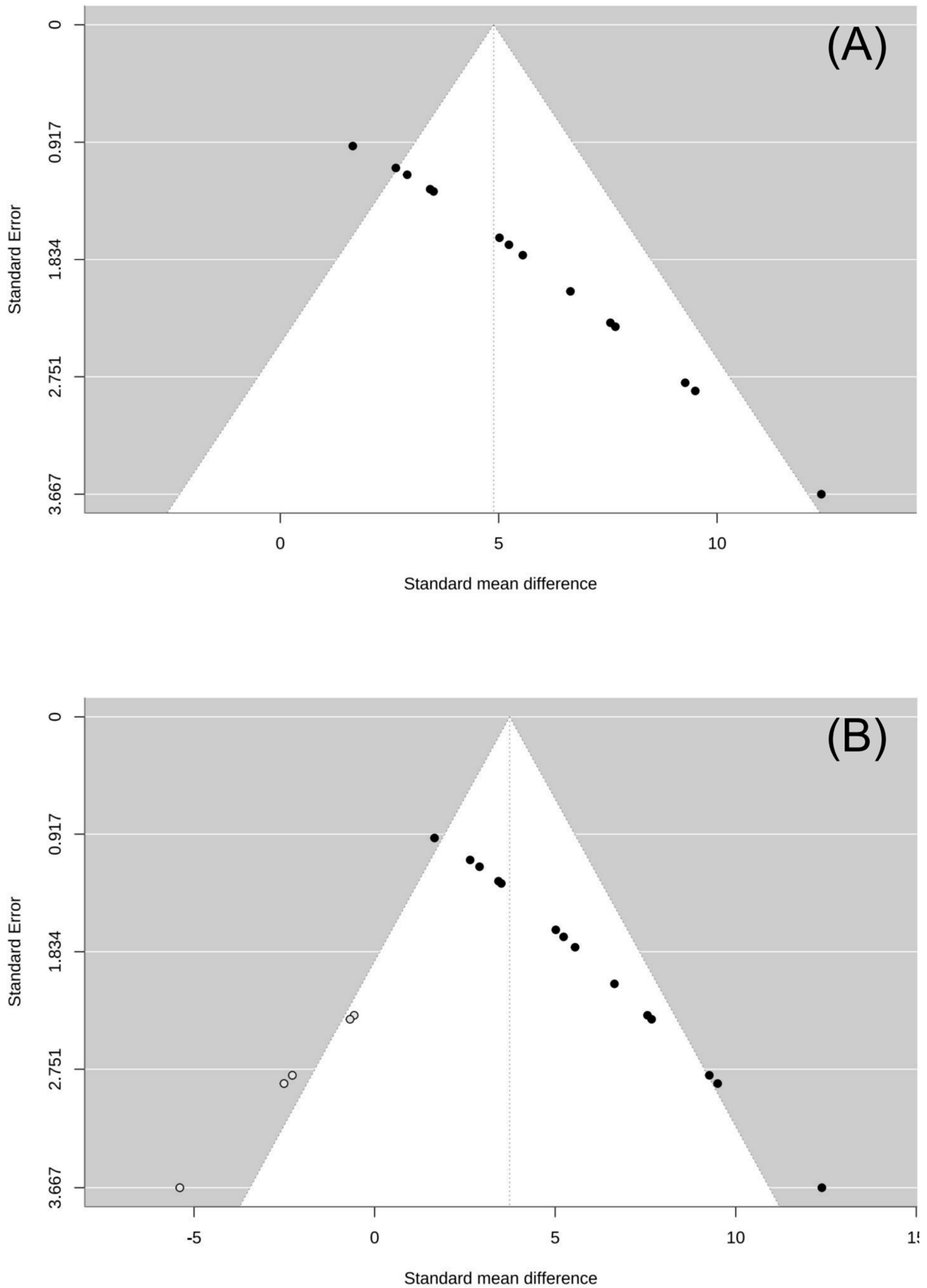

Figure S8. Evidence of publication (reporting) bias for protease. (a) Funnel plot of standardized mean difference; in the absence of bias, these points should be similar to a symmetrical inverted funnel shape. (b) Display the funnel plot of the missing studies supplemented by the 'trim and fill' method (shown in white); the white vertical line indicates the possible summary results if the theoretical missing studies are taken into account.

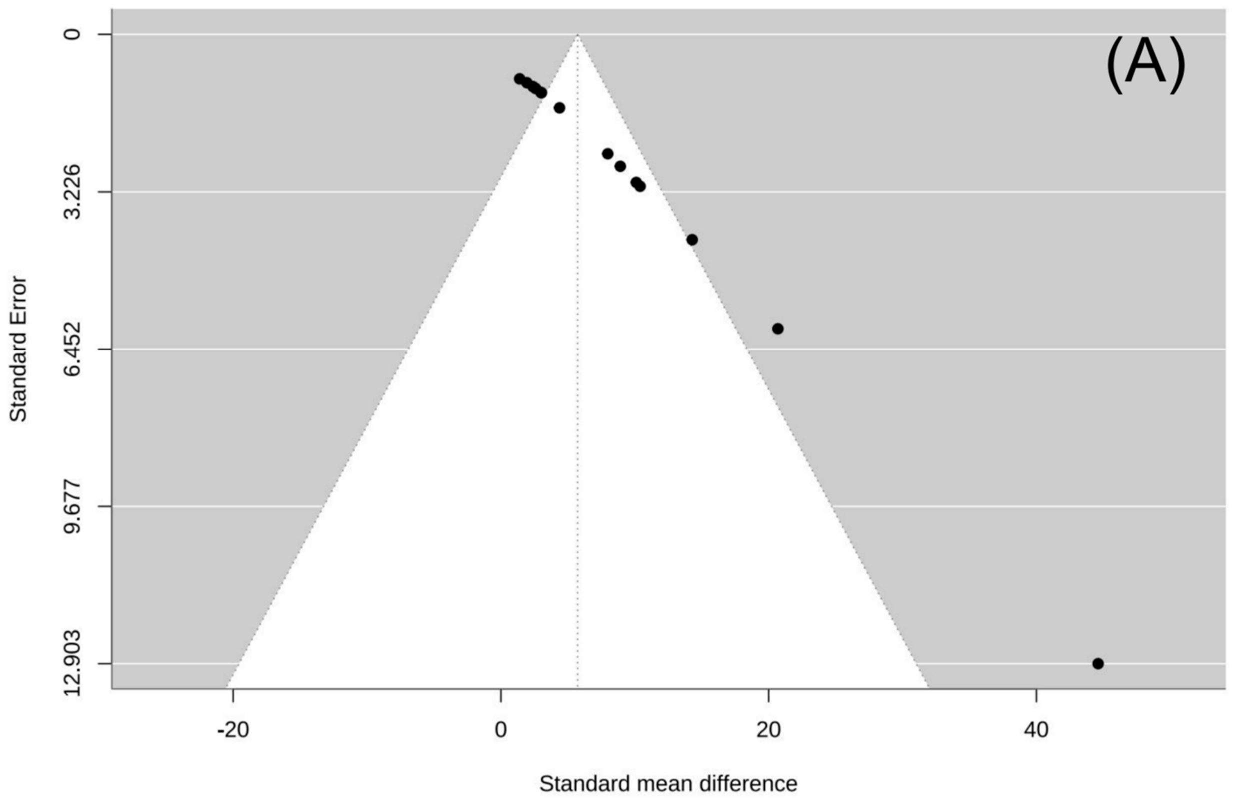

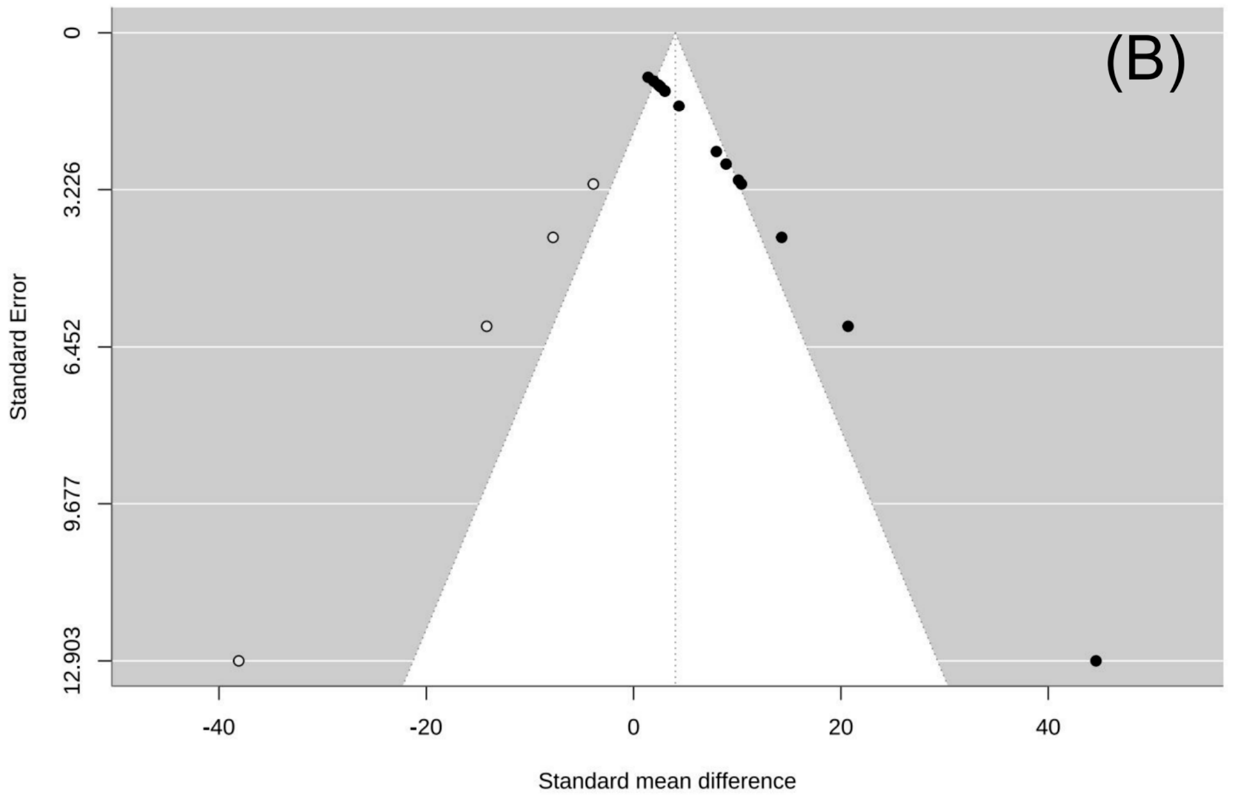

Figure S9. Evidence of publication (reporting) bias for amylase. (a) Funnel plot of standardized mean difference; in the absence of bias, these points should be similar to a symmetrical inverted funnel shape. (b) Display the funnel plot of the missing studies supplemented by the 'trim and fill' method (shown in white); the white vertical line indicates the possible summary results if the theoretical missing studies are taken into account.

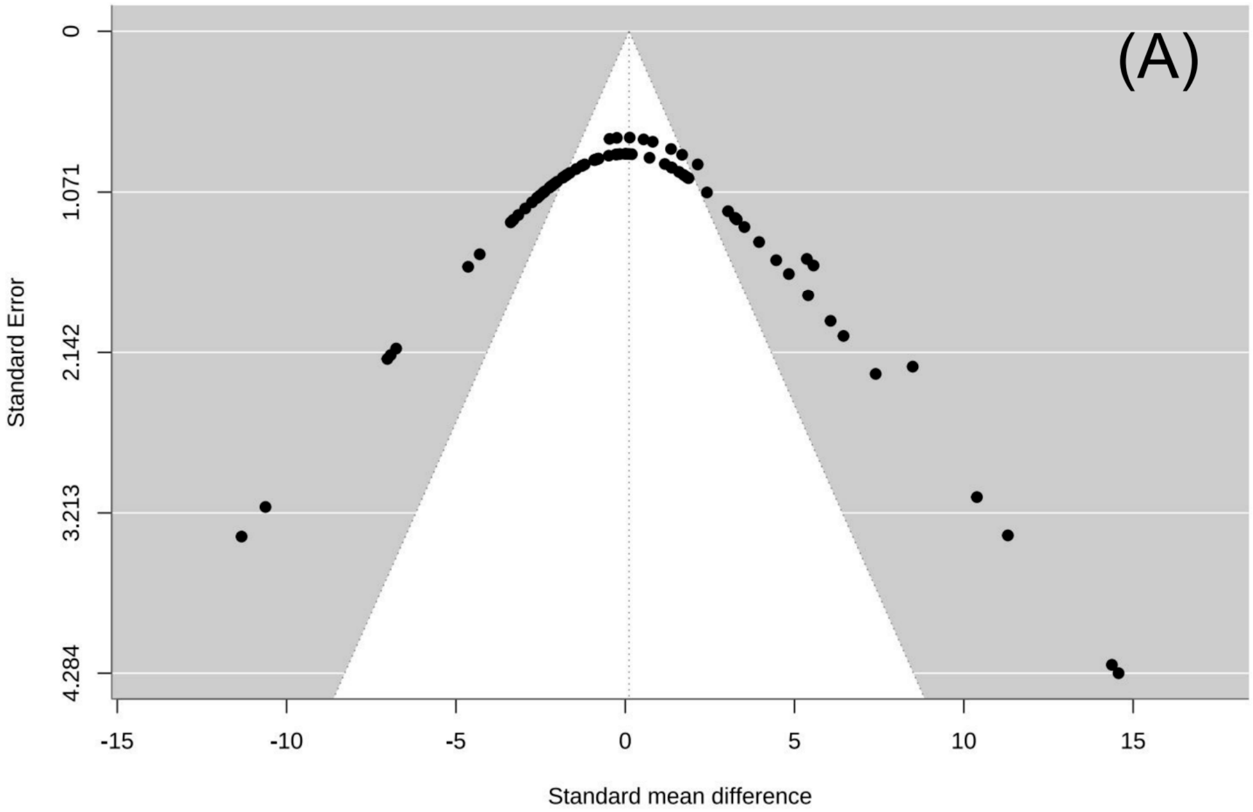

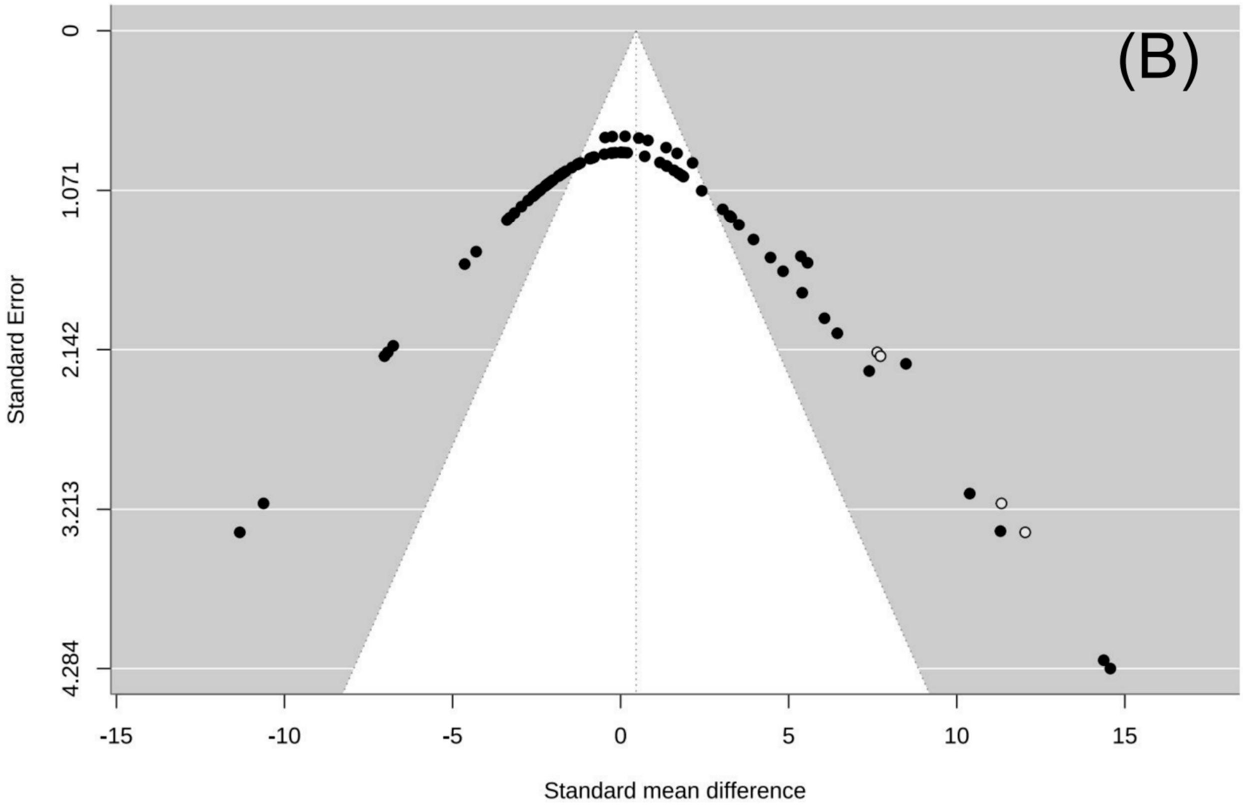

Figure S10. Evidence of publication (reporting) bias for SOD. (a) Funnel plot of standardized mean difference; in the absence of bias, these points should be similar to a symmetrical inverted funnel shape. (b) Display the funnel plot of the missing studies supplemented by the 'trim and fill' method (shown in white); the white vertical line indicates the possible summary results if the theoretical missing studies are taken into account.

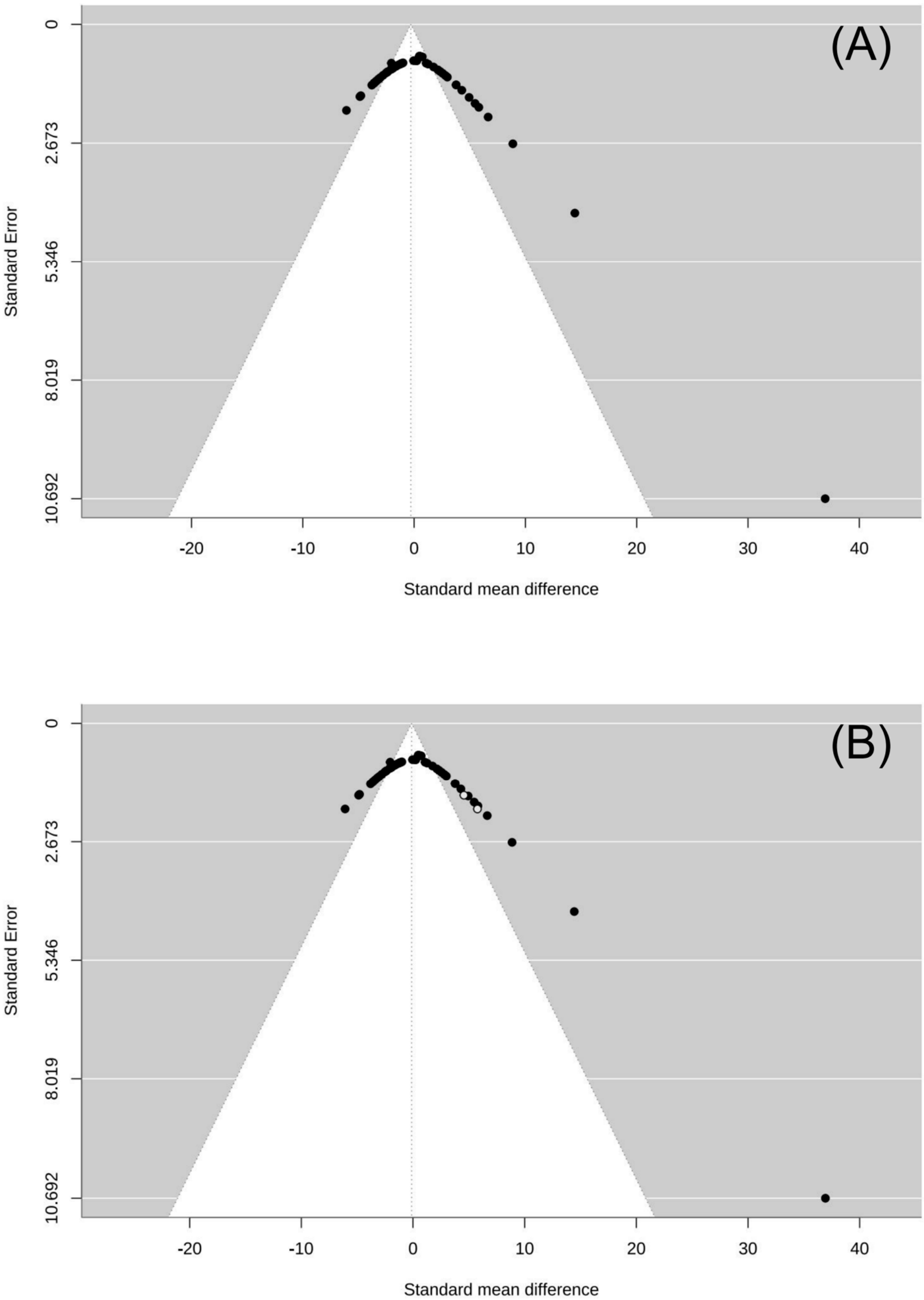

Figure S11. Evidence of publication (reporting) bias for CAT. (a) Funnel plot of standardized mean difference; in the absence of bias, these points should be similar to a symmetrical inverted funnel shape. (b) Display the funnel plot of the missing studies supplemented by the 'trim and fill' method (shown in white); the white vertical line indicates the possible summary results if the theoretical missing studies are taken into account.

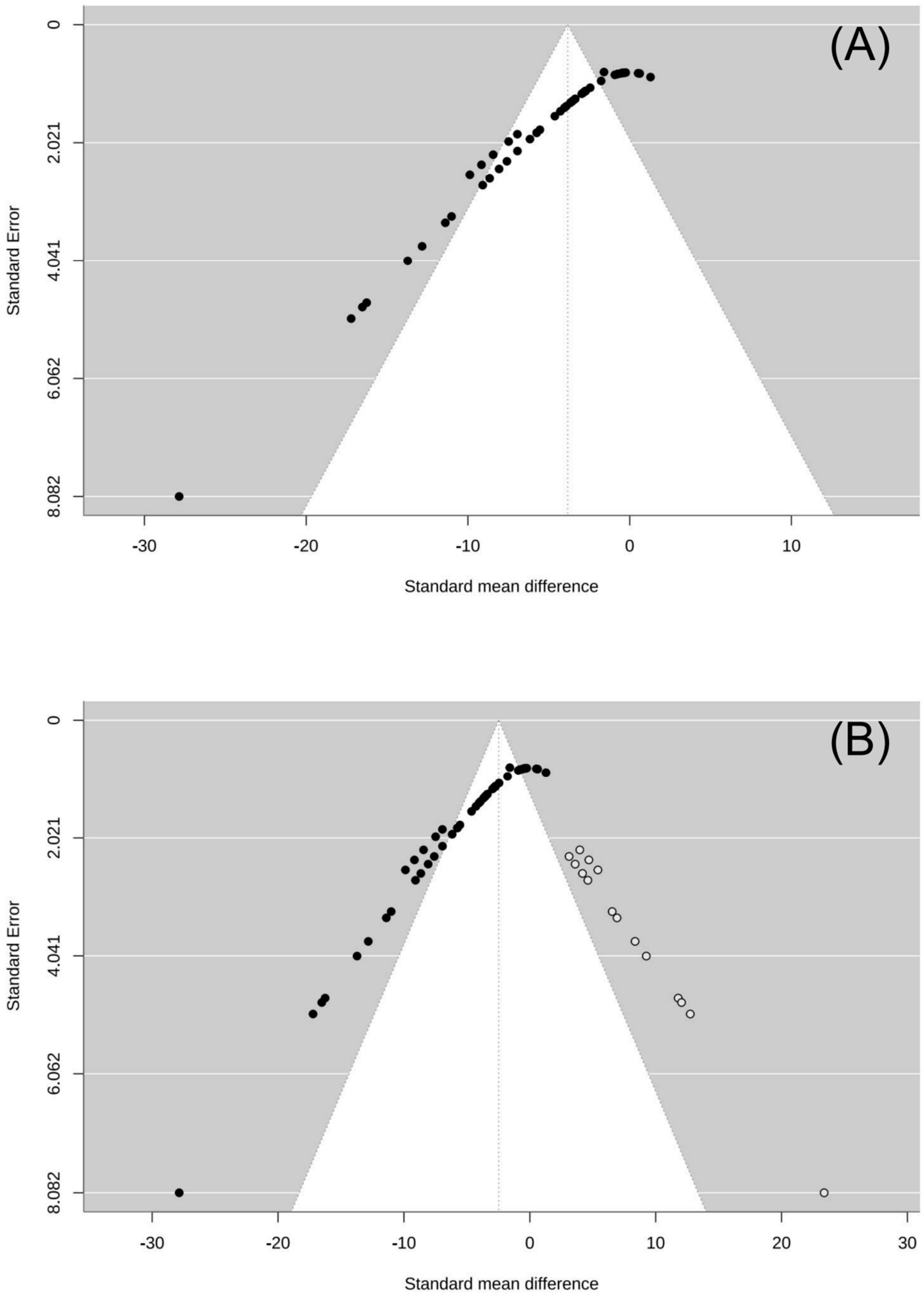

Figure S12. Evidence of publication (reporting) bias for MDA. (a) Funnel plot of standardized mean difference; in the absence of bias, these points should be similar to a symmetrical inverted funnel shape. (b) Display the funnel plot of the missing studies supplemented by the 'trim and fill' method (shown in white); the white vertical line indicates the possible summary results if the theoretical missing studies are taken into account.

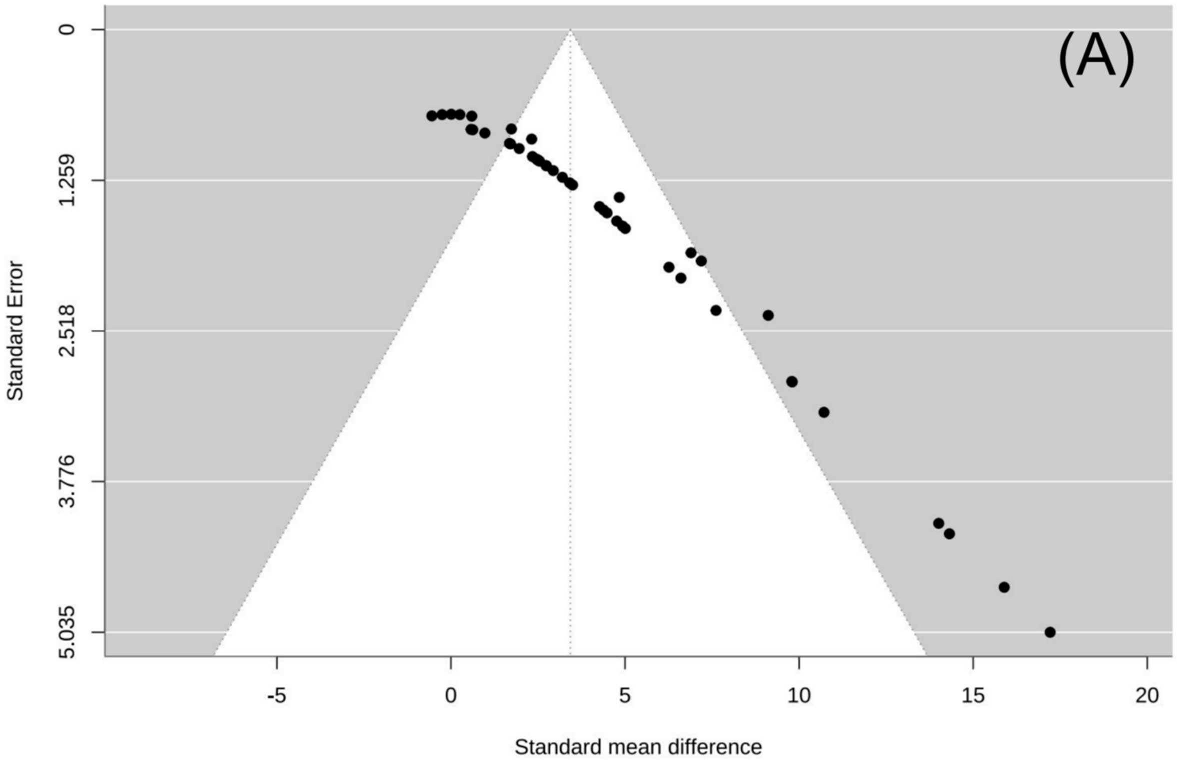

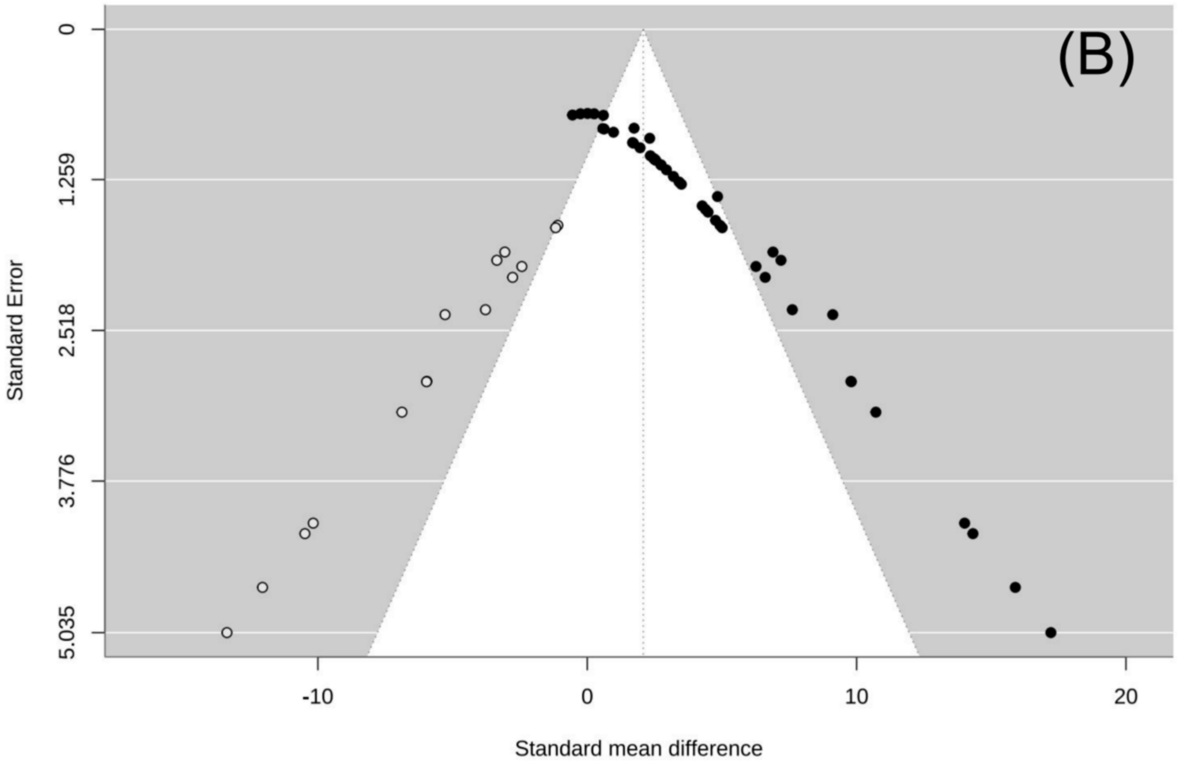

Figure S13. Evidence of publication (reporting) bias for T-AOC. (a) Funnel plot of standardized mean difference; in the absence of bias, these points should be similar to a symmetrical inverted funnel shape. (b) Display the funnel plot of the missing studies supplemented by the 'trim and fill' method (shown in white); the white vertical line indicates the possible summary results if the theoretical missing studies are taken into account.

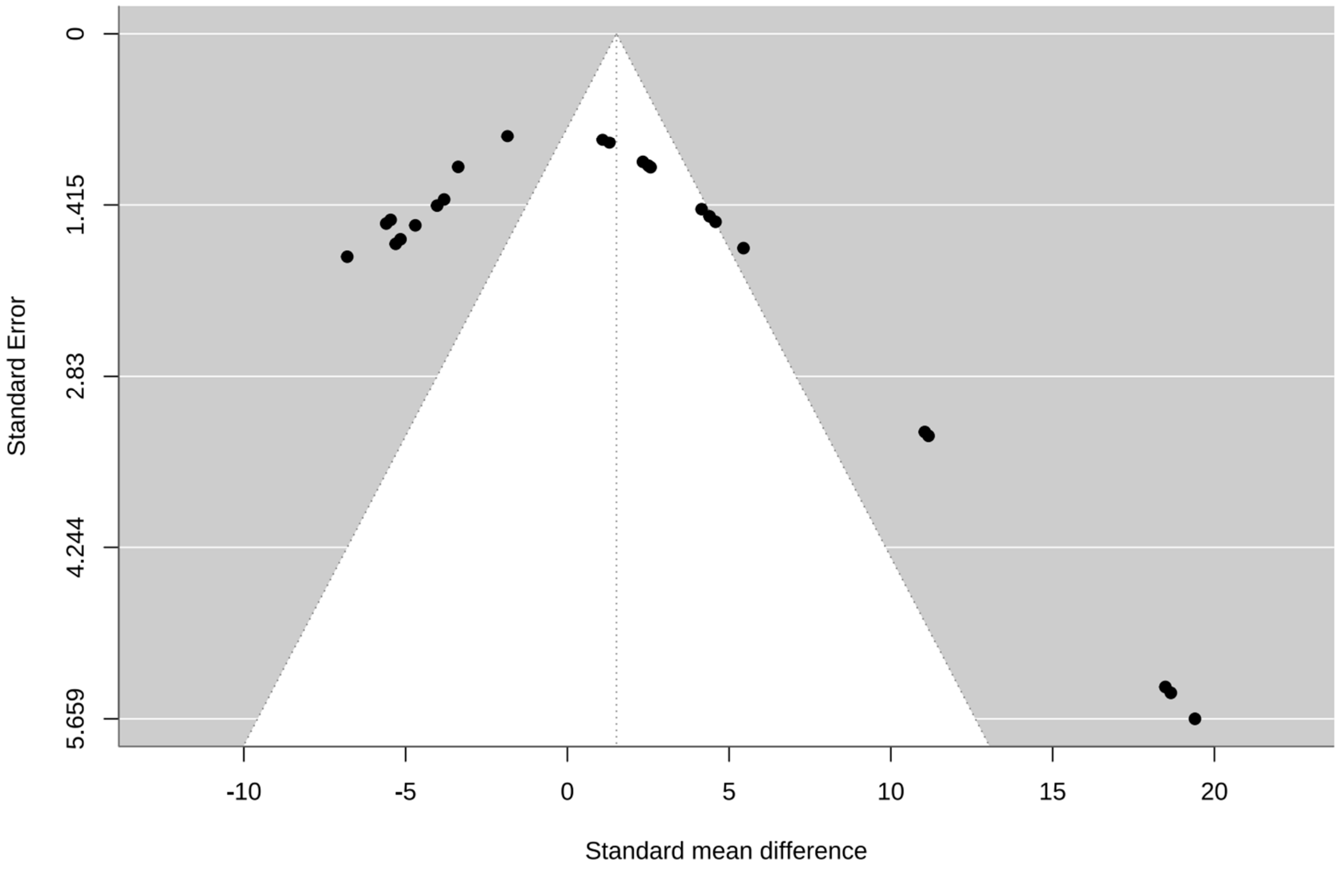

Figure S14. Evidence of publication (reporting) bias for GSH. Funnel plot of standardized mean difference; in the absence of bias, these points should be similar to a symmetrical inverted funnel shape.

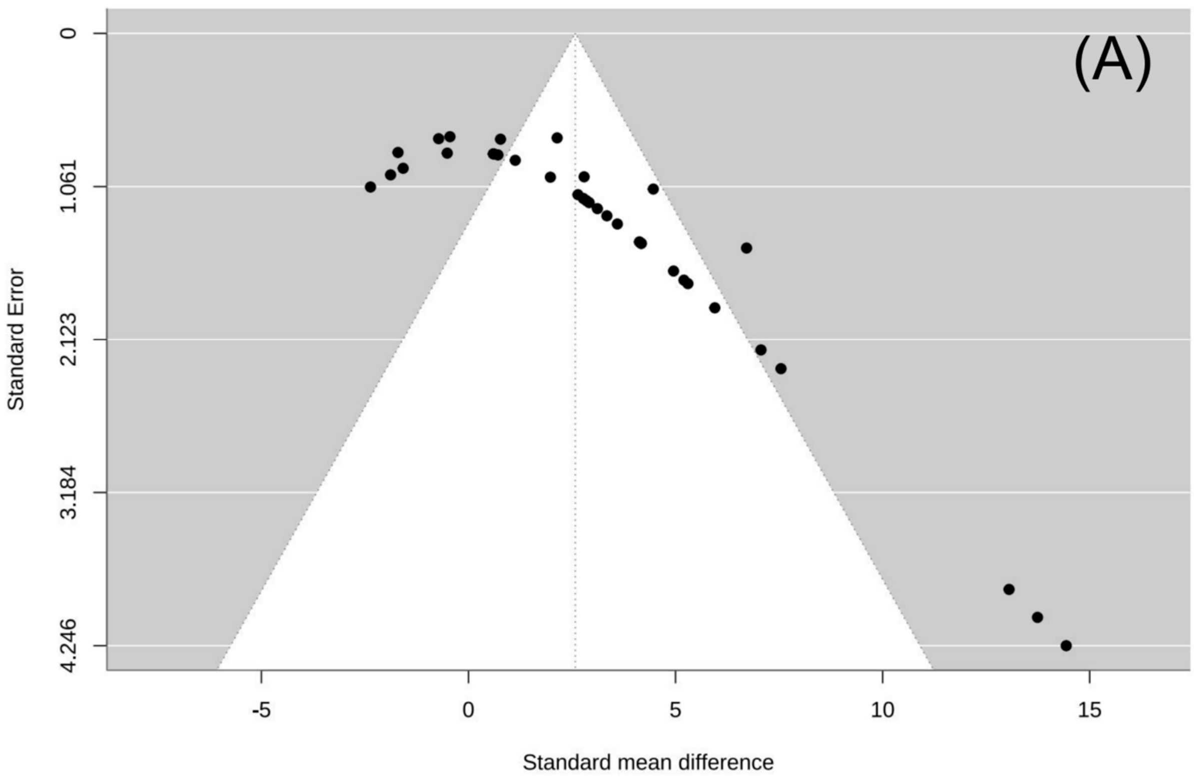

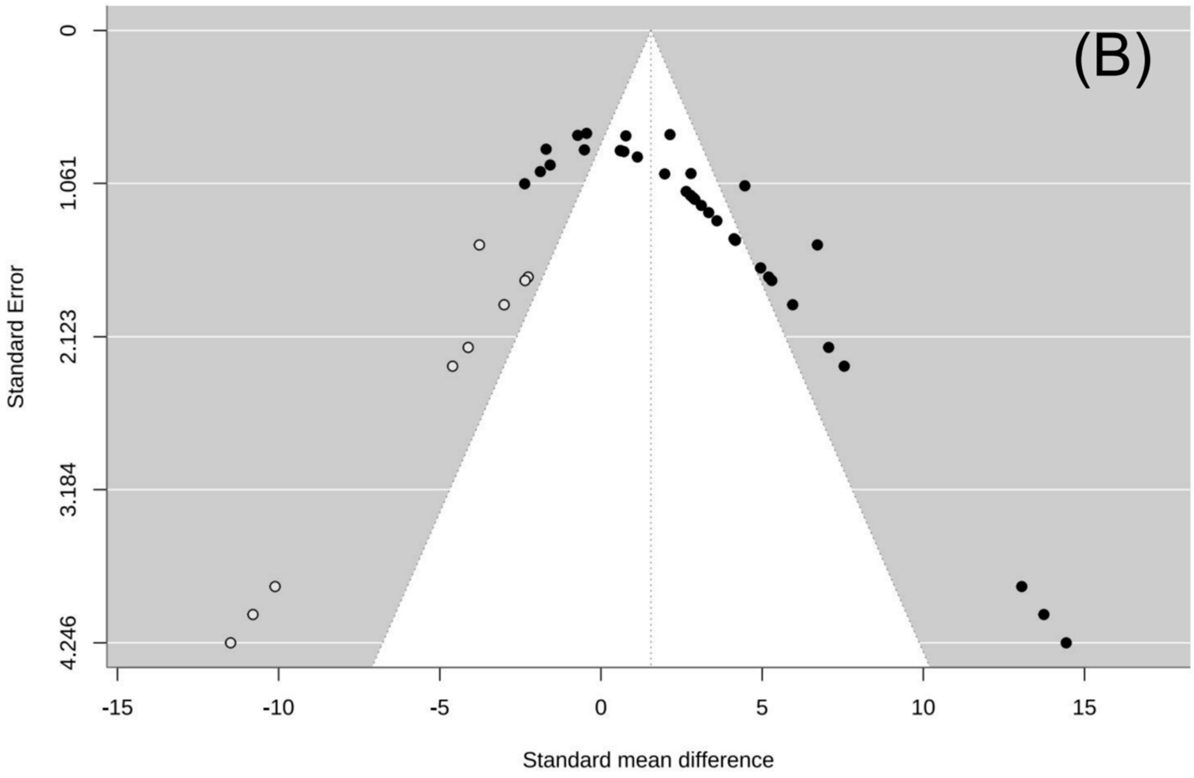

Figure S15. Evidence of publication (reporting) bias for GSH-Px. (a) Funnel plot of standardized mean difference; in the absence of bias, these points should be similar to a symmetrical inverted funnel shape. (b) Display the funnel plot of the missing studies supplemented by the 'trim and fill' method (shown in white); the white vertical line indicates the possible summary results if the theoretical missing studies are taken into account.

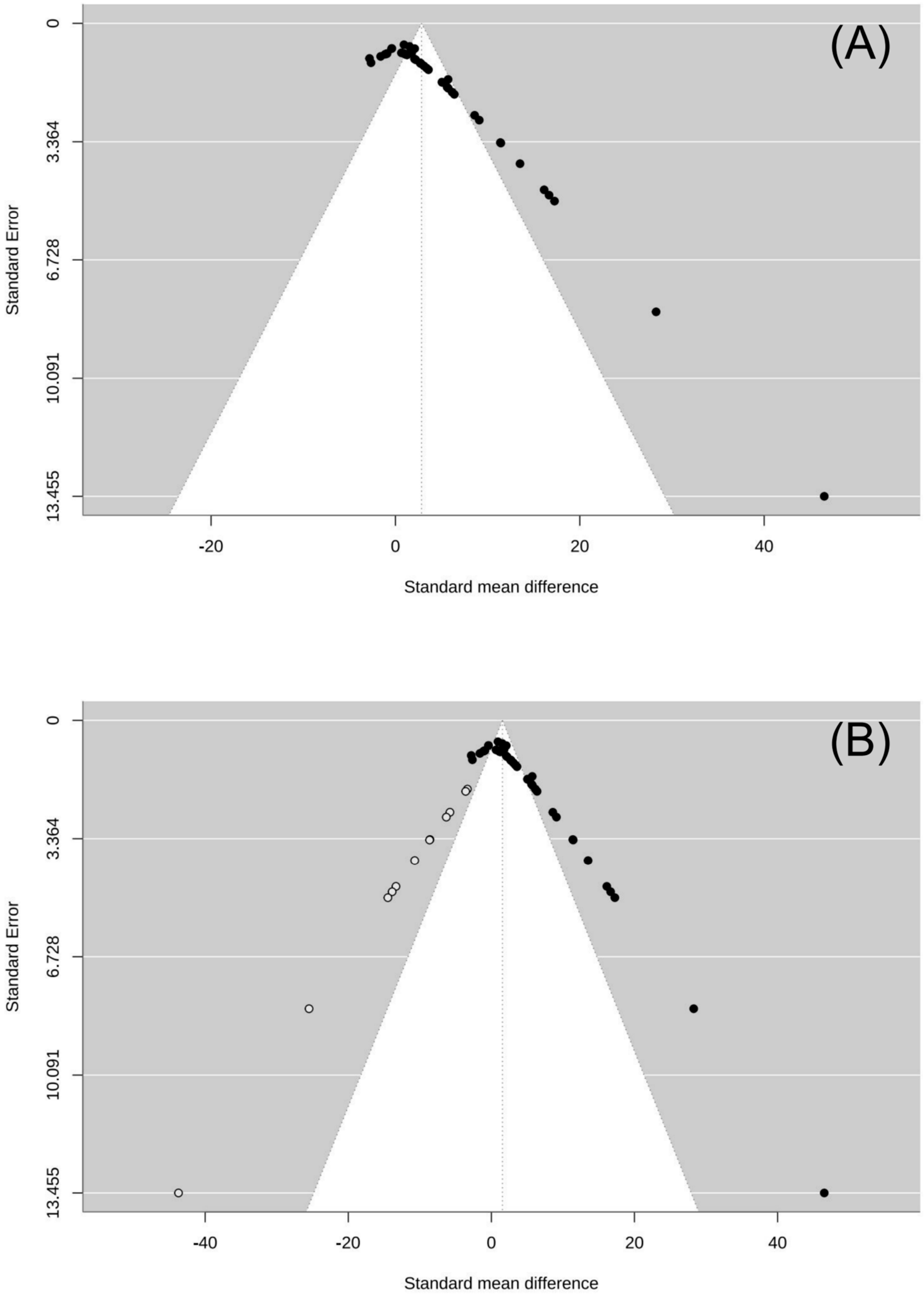

Figure S16. Evidence of publication (reporting) bias for lysozyme. (a) Funnel plot of standardized mean difference; in the absence of bias, these points should be similar to a symmetrical inverted funnel shape. (b) Display the funnel plot of the missing studies supplemented by the 'trim and fill' method (shown in white); the white vertical line indicates the possible summary results if the theoretical missing studies are taken into account.

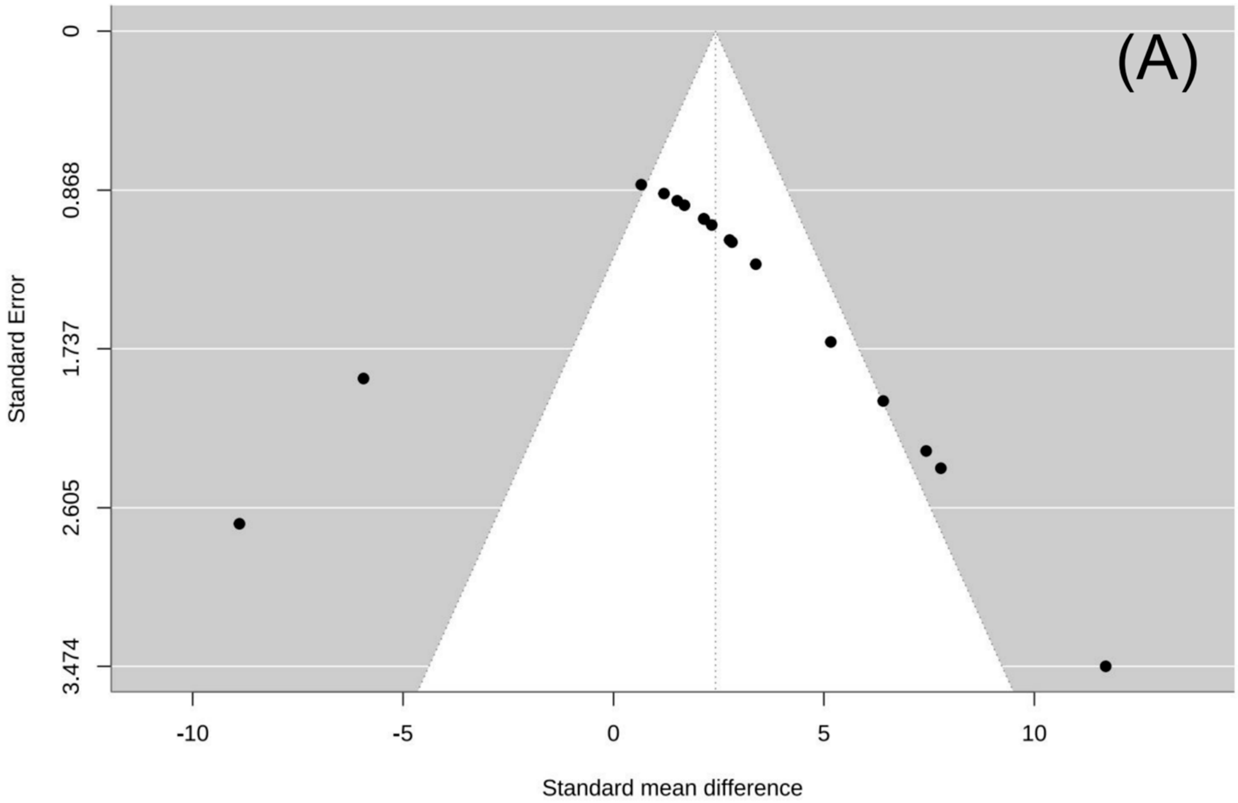

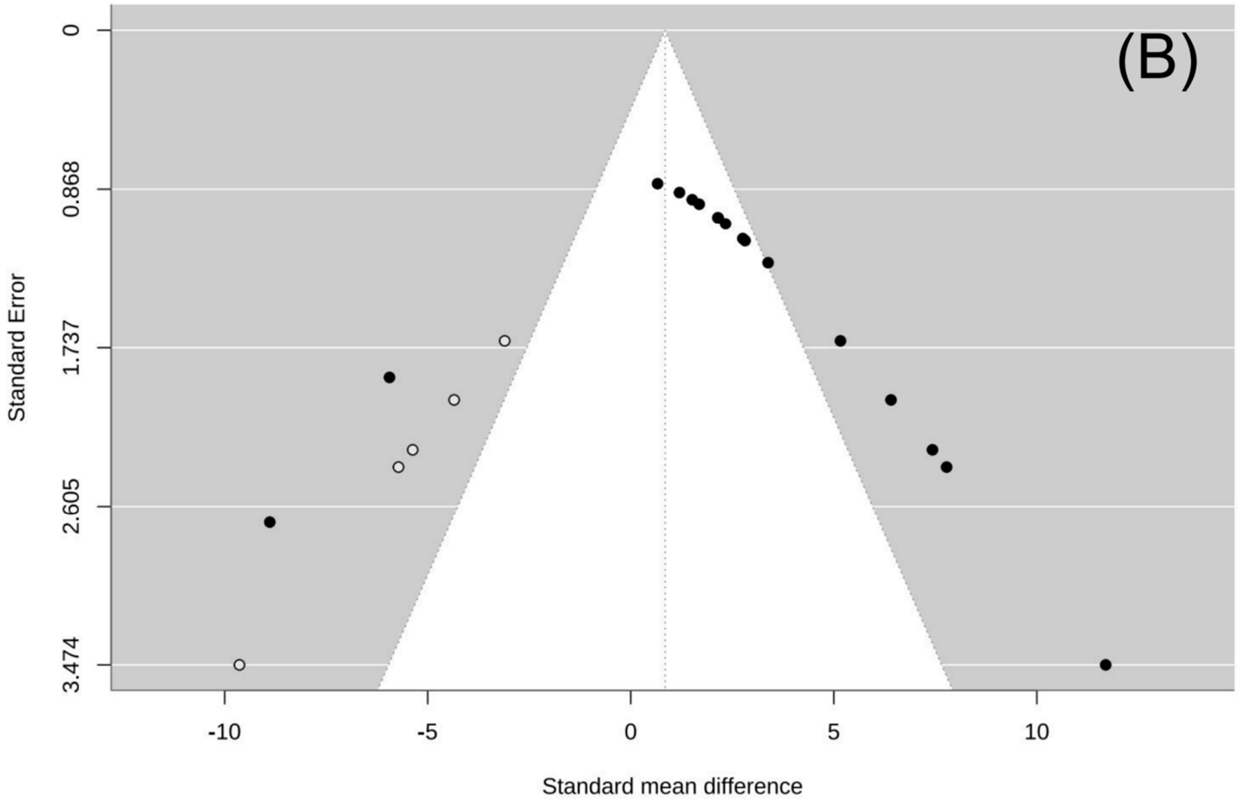

Figure S17. Evidence of publication (reporting) bias for Ig. (a) Funnel plot of standardized mean difference; in the absence of bias, these points should be similar to a symmetrical inverted funnel shape. (b) Display the funnel plot of the missing studies supplemented by the 'trim and fill' method (shown in white); the white vertical line indicates the possible summary results if the theoretical missing studies are taken into account.

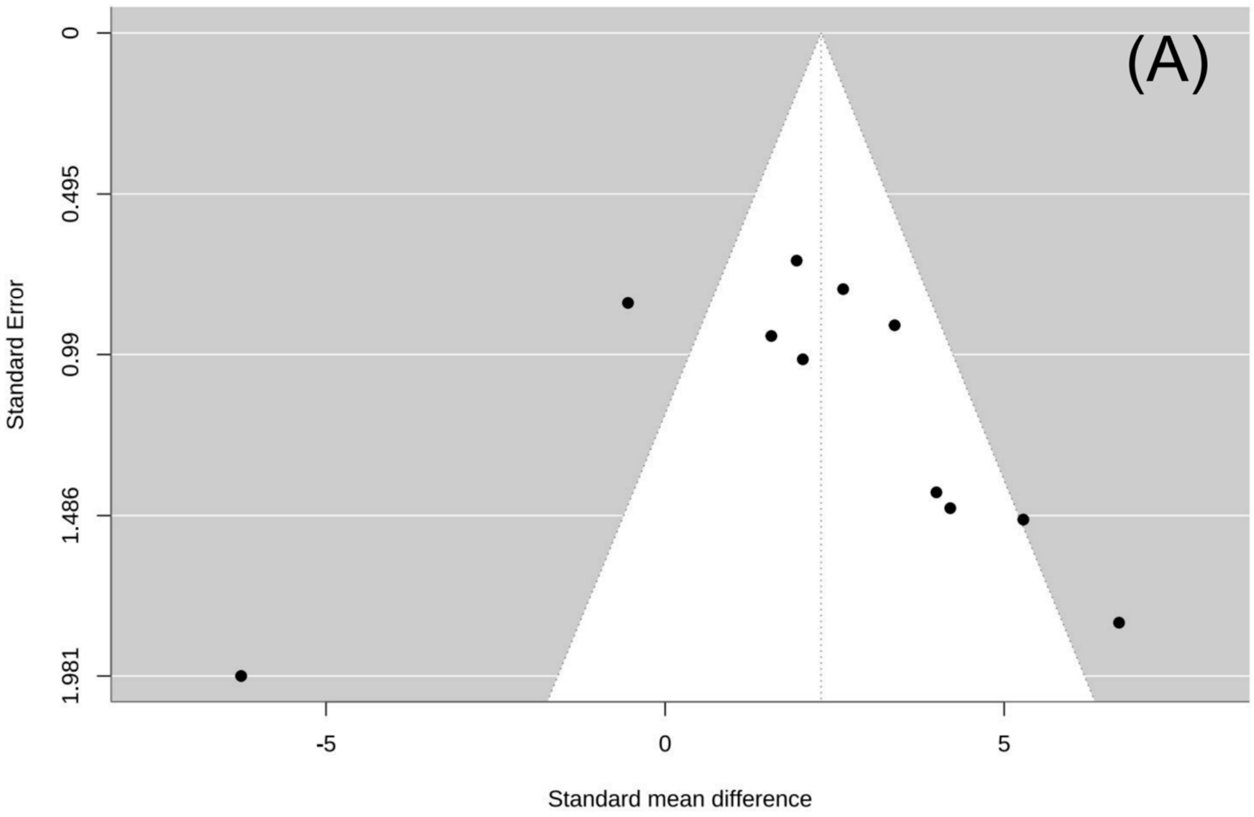

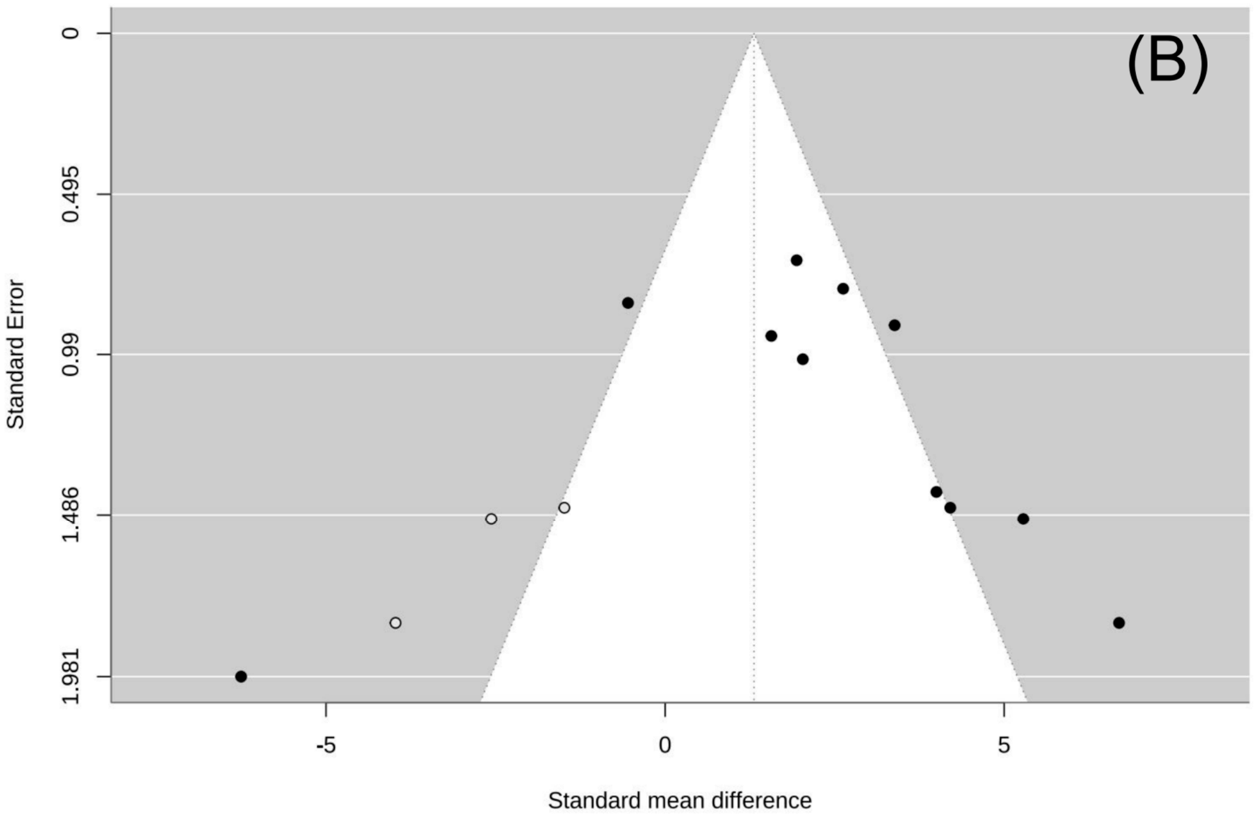

Figure S18. Evidence of publication (reporting) bias for C3. (a) Funnel plot of standardized mean difference; in the absence of bias, these points should be similar to a symmetrical inverted funnel shape. (b) Display the funnel plot of the missing studies supplemented by the 'trim and fill' method (shown in white); the white vertical line indicates the possible summary results if the theoretical missing studies are taken into account.

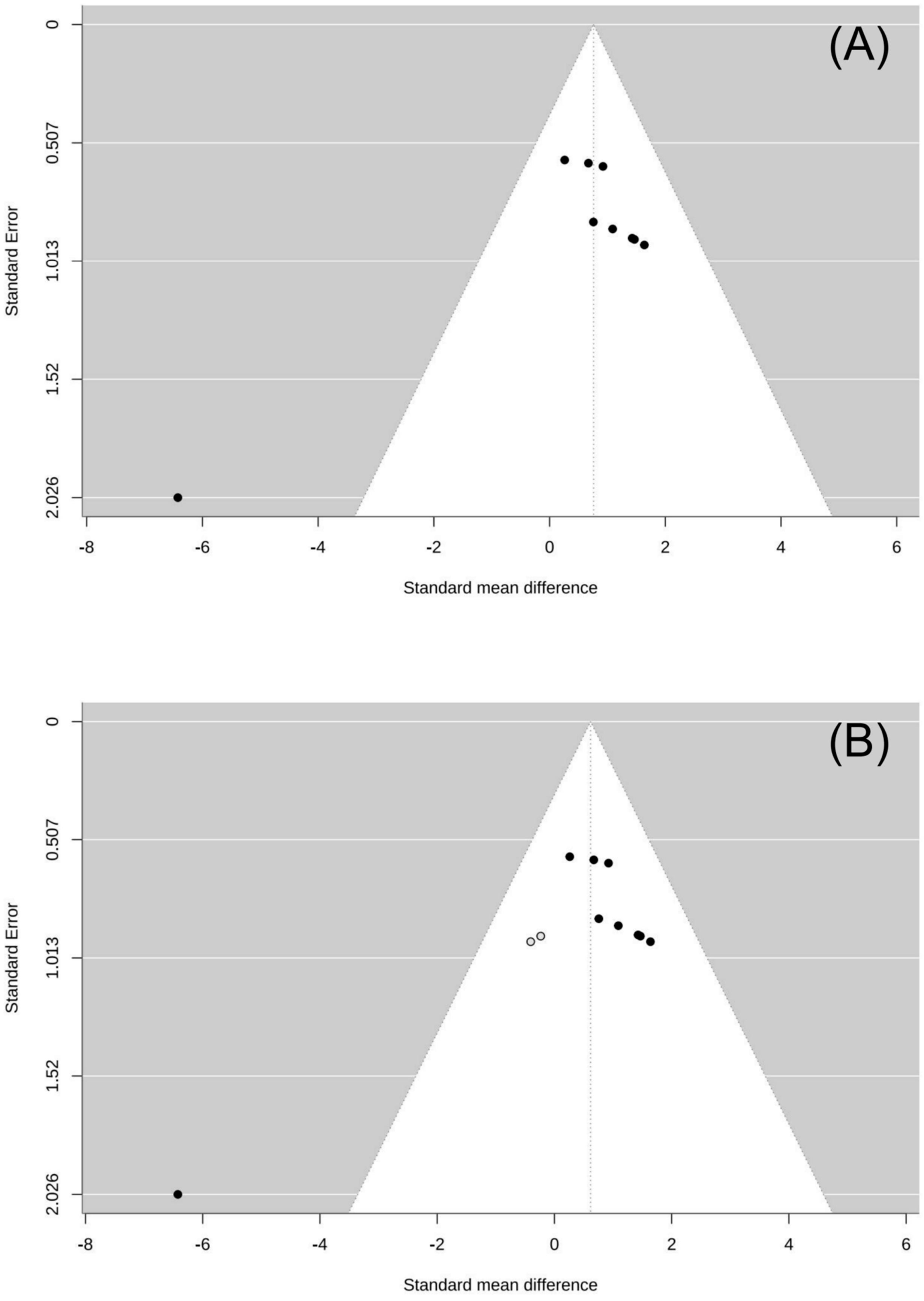

Figure S19. Evidence of publication (reporting) bias for C4. (a) Funnel plot of standardized mean difference; in the absence of bias, these points should be similar to a symmetrical inverted funnel shape. (b) Display the funnel plot of the missing studies supplemented by the 'trim and fill' method (shown in white); the white vertical line indicates the possible summary results if the theoretical missing studies are taken into account.

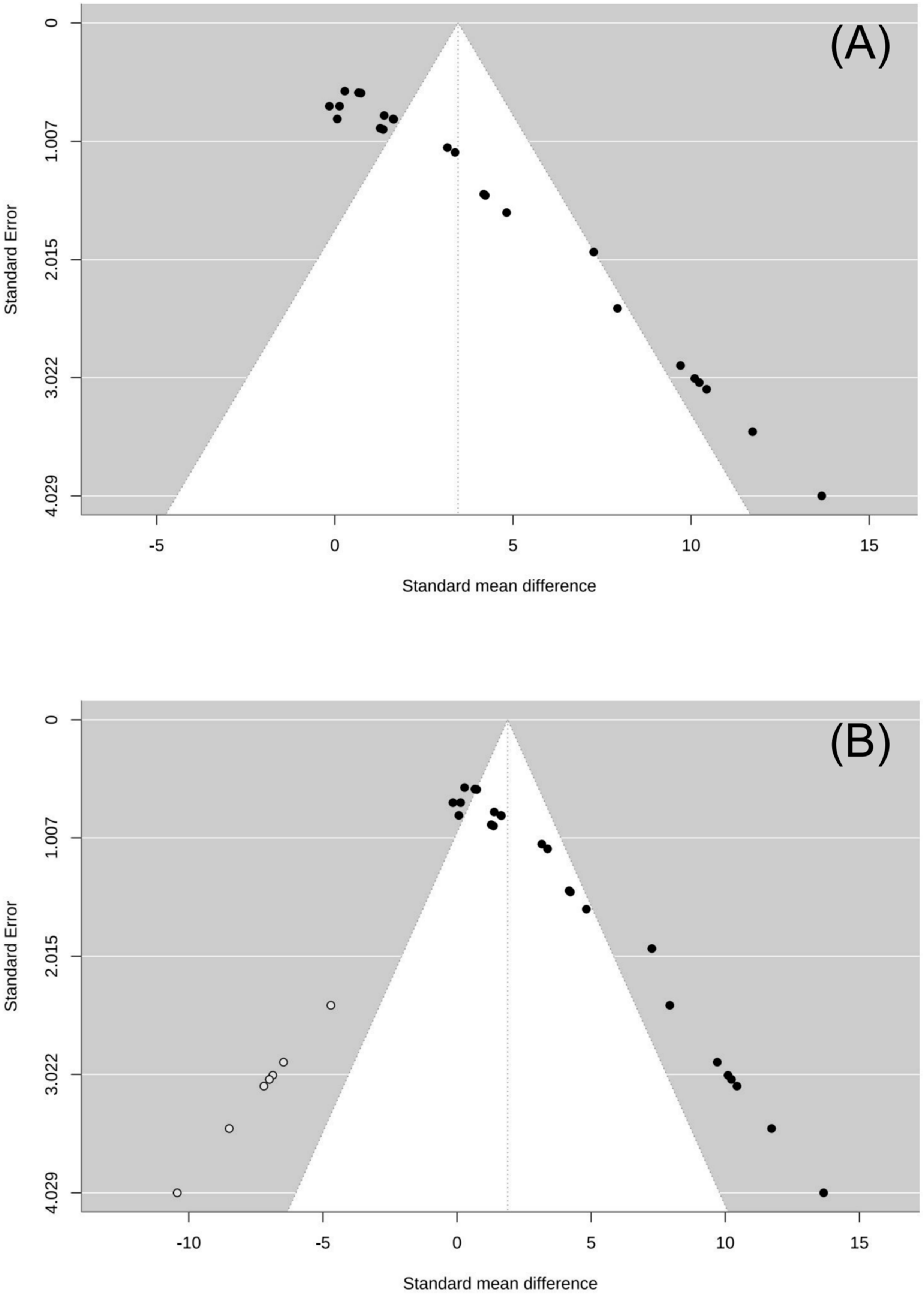

Figure S20. Evidence of publication (reporting) bias for ACP. (a) Funnel plot of standardized mean difference; in the absence of bias, these points should be similar to a symmetrical inverted funnel shape. (b) Display the funnel plot of the missing studies supplemented by the 'trim and fill' method (shown in white); the white vertical line indicates the possible summary results if the theoretical missing studies are taken into account.

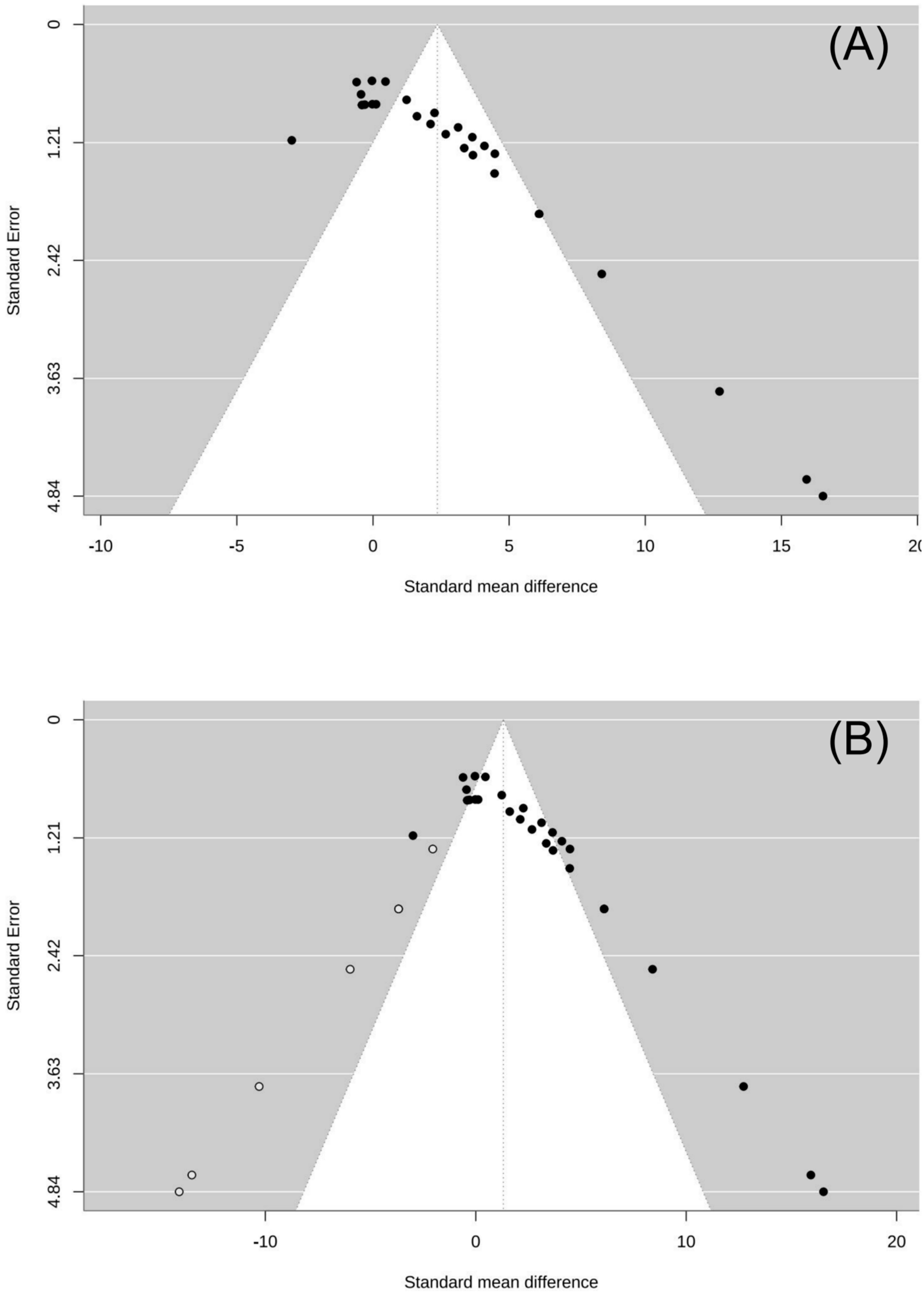

Figure S21. Evidence of publication (reporting) bias for AKP. (a) Funnel plot of standardized mean difference; in the absence of bias, these points should be similar to a symmetrical inverted funnel shape. (b) Display the funnel plot of the missing studies supplemented by the 'trim and fill' method (shown in white); the white vertical line indicates the possible summary results if the theoretical missing studies are taken into account.
